# Shedding Light on the Active Species in a Cobalt‐Based Covalent Organic Framework for the Electrochemical Oxygen Evolution Reaction

**DOI:** 10.1002/advs.202413555

**Published:** 2024-11-26

**Authors:** Pouya Hosseini, Andrés Rodríguez‐Camargo, Yiqun Jiang, Siyuan Zhang, Christina Scheu, Liang Yao, Bettina V. Lotsch, Kristina Tschulik

**Affiliations:** ^1^ Faculty of Chemistry and Biochemistry Analytical Chemistry II Ruhr‐Universität Bochum Universitätsstrasse150 44801 Bochum Germany; ^2^ Max Planck Institute for Sustainable Materials Max‐Planck‐Straße 1 40237 Düsseldorf Germany; ^3^ Nanochemistry Department Max Planck Institute for Solid State Research Heisenbergstraße 1 70569 Stuttgart Germany; ^4^ Department of Chemistry University of Stuttgart Pfaffenwaldring 55 70569 Stuttgart Germany; ^5^ State Key Laboratory of Luminescent Materials and Devices Institute of Polymer Optoelectronic Materials and Devices Guangdong Basic Research Center of Excellence for Energy and Information Polymer Materials South China University of Technology Guangdong 510640 China; ^6^ Department of Chemistry University of Munich (LMU) Butenandtstraße 5–13 81377 München Germany

**Keywords:** active site, catalysis, catalyst transformation, covalent organic framework, metal coordination

## Abstract

While considerable efforts have been devoted to developing functionalized covalent organic frameworks (COFs) as oxygen evolution electrocatalysts in recent years, studies related to the investigation of the true catalytically active species for the oxygen evolution reaction (OER) remain lacking in the field. In this work, the active species of a cobalt‐functionalized COF (TpBpy‐Co) is studied as electrochemical OER catalyst through a series of electrochemical measurements and post‐electrolysis characterizations. These results suggest that cobalt oxide‐based nanoparticles are formed in TpBpy‐Co from Co(II) ions coordinated to the COF backbone when exposing TpBpy‐Co to alkaline media, and these newly formed nanoparticles serve as the primary active species for oxygen evolution. The study thus emphasizes that caution is warranted when assessing the catalytic activity of COF electrocatalysts, as the pristine COF may act as the pre‐catalyst, with the active species forming only under catalyst operating conditions. Specifically, strong coordination between COFs and metal centers under electrochemical operation conditions is crucial to avoid unintended transformation of COF electrocatalysts. This work thus contributes to the rational development of earth‐abundant COF OER catalysts for the production of green hydrogen from renewable resources.

## Graphical abstract

We study the catalytically active species of a cobalt‐based covalent organic framework (TpBpy‐Co) for the electrochemical oxygen evolution reaction (OER). Our results reveal that cobalt‐based nanoparticles, very active for OER, are formed when exposing TpBpy‐Co to an alkaline electrolyte and OER conditions, demonstrating the importance of catalytic site studies for developing new COF OER catalysts.

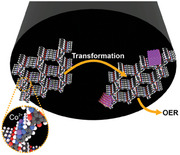

## 1 Introduction

Developing efficient catalysts to oxidize water to oxygen is one of the central tasks in electrocatalysis, as the oxygen evolution reaction (OER) is the common anodic reaction in many electrochemical energy conversion systems producing renewable fuels or value‐added‐chemicals. Amongst the most important examples are water electrolysis, electrochemical CO_2_ or N_2_ reduction, and metal‐air batteries.^[^ ^1^ ^]^ Given the sluggish kinetics of the four‐electron transfer process, it has proven particularly challenging to achieve high performance OER catalysts. While noble metals (iridium or ruthenium) based compounds have exhibited promising catalytic activities, the scarcity of noble metals limits their economically viable application. Hence, there is an obvious need to develop earth‐abundant metal‐based OER catalysts. Molecular catalysts, comprising first row transition metals, have shown the capability to act as competitive OER catalysts and therefore attracted increasing attention in recent years.^[^ ^2^, ^3^ ^]^ Moreover, engineering the ligands of molecular catalysts can endow atomistic tunability for catalytic activity and further establish structure‐function relationships at the molecular level. For instance, Masaok et al. designed a pentanuclear iron complex, which shows a high turnover frequency of 1900 s^−1^ for water oxidation, and subsequently introducing substituents on the ligand is used to reduce the overpotential of this complex.^[^ ^4^, ^5^ ^]^ However, the downside of molecular catalysts is that they may lose their integrity under operational conditions and irreversibly transform into metal‐based nanoparticles, which then act as the true catalysts for OER.^[^ ^6^ ^]^ More generally, the picture of a catalyst as a static particle with “frozen” active sites has been increasingly challenged over the past years. Instead, evidence for highly dynamic catalyst structures has been emerging, which – while challenging to analyze – offer new perspectives for catalyst reconfiguration, rejuvenation, and dynamic catalysis.^[^ ^7^, ^8^, ^9^, ^10^ ^]^ Therefore, in both heterogeneous and molecular catalysis great efforts have been made to understand whether the catalyst acts as a pre‐catalyst or the actual catalytic species.^[^ ^6^, ^11^, ^12^, ^13^, ^14^, ^15^, ^16^, ^17^, ^18^, ^19^ ^]^ A classic example addresses cobalt‐based polyoxometalates (Co_4_POM). A series of reports revealed that Co_4_POM can decompose and form amorphous CoO_x_ on the electrode when working as homogeneous OER catalyst in pH 8 sodium phosphate buffer solutions, while both Co_4_POM and CoO_x_ can be active for electrochemical or photocatalytic oxygen evolution under suitable conditions.^[^ ^11^, ^12^, ^13^, ^20^, ^21^ ^]^ Another example are tetranuclear manganese clusters, doped into a Nafion matrix to form a heterogenized catalyst for photoelectrochemical oxygen evolution.^[^ ^22^ ^]^ These manganese clusters were identified as pre‐catalysts for OER, while manganese oxide, obtained in‐situ from decomposition of these manganese clusters under operational condition, are the actual catalytic species.^[^ ^15^, ^23^ ^]^ Given the importance of understanding the role of molecular catalysts, their structural integrity under operational conditions and the true active species have to be carefully evaluated, as emphasized in recent reviews on molecular catalysts.^[^ ^2^, ^3^ ^]^

**Figure 1:**
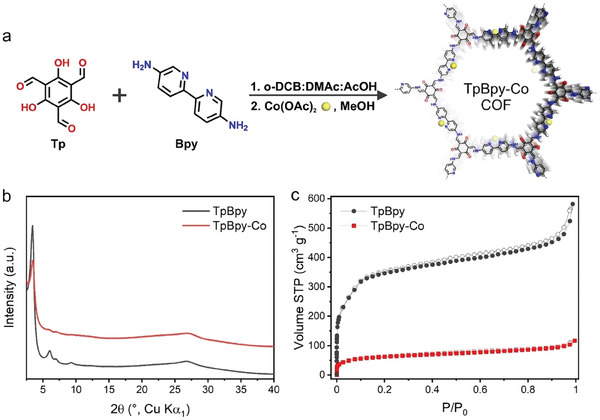
a) Schematic representation of the synthesis of TpBpy‐Co COF; b) PXRD patterns (Cu Kα_1_) of TpBpy and TpBpy‐Co; c) N_2_ adsorption (filled) and desorption (empty) isotherm profiles at 77 K.

Covalent organic frameworks are a class of extended crystalline polymers which are built by linking organic building units with covalent bonds into extended frameworks with permanent porosity.^[^ ^24^ ^]^ By selecting suitable linkage‐types and building units, COFs can be tailored to provide a large number of identical and structurally defined catalytically active sites, which are well accessible due to the high porosity of COFs. Their molecularly precise character, paired with structural porosity and robustness, sets COFs apart from conventional molecular catalysts. Therefore, COFs have been intensely studied as heterogeneous electrocatalysts in recent years.^[^ ^25^ ^]^ It is noteworthy that although the pristine COFs are prone to hydrolytic decomposition, chemical and structural stability of COFs has significantly been enhanced in recent years. In fact, state‐of‐the‐art COFs, such as β‐ketoenamine‐linked or post‐synthetically locked COFs, can withstand harsh aqueous electrolytes (strongly acidic or alkaline), providing a solid basis for applying COFs in electrocatalysis.^[^ ^26^, ^27^ ^]^ So far, a number of COFs comprised of bipyridine, pyrimidine, tetrazole, or sulfonate, etc. as building blocks were employed to incorporate first row transition metals, and were reported to be catalytically active for electrochemical OER.^[^ ^28^, ^29^, ^30^, ^31^, ^32^, ^33^, ^34^, ^35^, ^36^ ^]^ Previous studies on the catalytic mechanism suggested that metal atoms coordinated to the COF backbone are critical for the COF's catalytic activity, while a permanent change in oxidation state of the metal after OER catalysis has been reported.^[^ ^29^ ^]^ However, an understanding of the true catalytically active species in metal ion‐containing COFs is still largely lacking. Considering the crucial role of identifying the active species for the future design of COF electrocatalysts, herein, we report the first systematic investigation of the catalytically active species in COF OER electrocatalysts, by studying the catalytic mechanism of a previously reported COF OER catalyst based on the β‐ketoenamine‐linkage, TpBpy‐Co (**Figure** **1**a).^[^ ^28^, ^29^ ^]^ Contrary to several reports suggesting the high stability of COFs under OER conditions, we demonstrate that the coordination between the cobalt ion and TpBpy is unstable in alkaline electrolyte. This instability leads to the rapid transformation of cobalt ions into heterogeneous cobalt hydroxide species, which further evolve under OER conditions to provide the active sites for electrocatalytic OER.

## 2 Results and Discussion

TpBpy COF was prepared by condensation of 1,3,5‐trifloroglucinol (Tp) and 5,5′‐diamino‐2,2′‐bipyridine (Bpy) via a Schiff‐base reaction according to the reported solvothermal approach (Figure 1a).^[^ ^28^ ^]^ The resulting TpBpy‐COF was immersed in methanolic cobalt(II) acetate solution to load Co(II) ions on the bipyridine units and obtain cobalt loaded TpBpy COF (TpBpy‐Co). Synthesis details of TpBpy and TpBpy‐Co are summarized in the Supporting Information. To confirm the success of Co(II) ion loading, inductively coupled plasma optical emission spectroscopy (ICP‐OES) analysis and UV–vis diffuse reflectance spectra (UV–vis DRS) were performed. The cobalt amount in TpBpy‐Co was determined to be 11 wt.% by ICP‐OES analysis, in agreement with previously reported work.^[^ ^28^ ^]^ In addition, it is known that after introducing Co(II) ions on bipyridine ligands, the metal‐ligand charge transfer results in the appearance of low energy transitions in the UV–vis absorption spectrum.^[^ ^28^, ^37^ ^]^ As shown in the UV–vis DRS (Figure S2, Supporting Information), a clear red shift of the absorption band is observed for TpBpy‐Co, and its optical bandgap slightly decreases by 0.08 eV, further confirming the Co(II) ion loading on bipyridine units (Figure S3, Supporting Information). Powder X‐ray diffraction (PXRD) measurements were performed to investigate the crystallinity of TpBpy and TpBpy‐Co. As shown in Figure 1b, TpBpy exhibits a pronounced diffraction peak at 2*θ*  =  3.42° corresponding to the 100 plane, and other clearly visible diffraction peaks at 2*θ*  =  6.02, 6.92, 9.27, 26.70°, assigned as 110, 200, 220, 001 planes, respectively. The observed diffraction peaks match well with calculated patterns obtained using the eclipsed stacking model (Figure S4, Supporting Information). In comparison, while TpBpy‐Co retains the 100 peak at 2*θ*  =  3.42°, higher order diffraction signals broaden and diminish, suggesting decreased crystallinity due to the cobalt loading. This is likely caused by the COF layer distortion associated with the presence of charged cobalt ions, which introduces a repulsive interaction between the COF layers.^[^ ^38^ ^]^ The porosity of TpBpy and TpBpy‐Co was investigated by measuring their nitrogen sorption isotherms at 77 K. The Brunauer–Emmett–Teller surface areas are calculated to be 1331 and 228 m^2^ g^−1^ for TpBpy and TpBpy‐Co (Figures S5 and S6, Supporting Information), respectively. In addition, TpBpy‐Co shows a reduced pore size (2.0 nm) compared to pristine TpBpy (2.3 nm) (Figures S7 and S8, Supporting Information). Therefore, the 5‐fold lower BET surface area is likely attributable to the combined effects of increased molecular weight of cobalt‐loaded TpBpy‐Co and its reduced crystallinity, resulting in less accessible pores.

**Figure 2:**
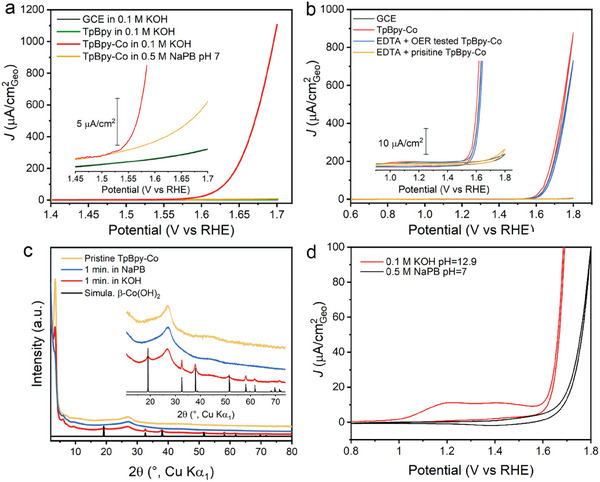
a) Linear sweep voltammetry curves of bare GCE, TpBpy, and TpBpy‐Co in 0.1 m KOH (pH 12.9), and TpBpy‐Co in NaPB at pH 7 at a scan rate of 10 mV s^−1^; b) CV curves of TpBpy‐Co, EDTA + OER tested TpBpy‐Co, and EDTA + pristine TpBpy‐Co in 0.1 m KOH at a scan rate of 10 mV s^−1^; c) PXRD patterns (Cu Kα_1_) of pristine TpBpy‐Co and electrolyte soaked TpBpy‐Co. d) CV curves of TpBpy‐Co in 0.1 m KOH and 0.5 m NaPB (pH 7) at a scan rate of 100 mV s^−1^.

The electrochemical oxygen evolution catalytic activity of TpBpy‐Co was examined in both neutral and alkaline aqueous electrolytes, which have also been used in previous reports,^[^ ^28^, ^29^ ^]^ with a rotating disk electrode setup (**Figure** **2**a). A glassy carbon electrode (GCE) with an area of 0.196 cm^2^, a graphite rod, and a reversible hydrogen electrode (RHE) were used as a working, counter, and reference electrode, respectively. Since Fe impurities have been reported to significantly influence OER activity in alkaline electrolytes,^[^ ^39^ ^]^ particular attention was paid to avoid Fe contamination in the electrolyte (details shown in Supporting Information). In neutral sodium phosphate buffer electrolyte (0.5 m NaPB, pH 7.0), no obvious current density increase compared to a bare GCE is observed for TpBpy‐Co by sweeping the potential up to +1.7 V versus RHE, indicating poor catalytic activity. By contrast, in alkaline electrolyte (0.1 m KOH (aq), pH 12.9), TpBpy‐Co shows a significant catalytic current for water oxidation, and the current density increases by ≈3 orders of magnitude compared to the current density recorded for an unmodified GCE at +1.7 V versus RHE. The Tafel slope in the kinetically controlled region^[^ ^40^ ^]^ (see details in Supporting Information) of TpBpy‐Co in 0.1 m KOH is calculated to be ≈50 mV dec^−1^ (Figure S9, Supporting Information), in agreement with literature results.^[^ ^29^ ^]^ The striking performance difference with varying electrolyte pH motivated us to explore the catalytically active species of TpBpy‐Co for OER. Accordingly, we initially examined whether TpBpy‐Co is the active OER catalyst or whether it acts as a pre‐catalyst, forming aqueous cobalt ions or cobalt‐based nanoparticles that act as the true active OER catalysts under operation conditions.^[^ ^20^, ^41^, ^42^ ^]^ To exclude aqueous cobalt ions as the catalytically active species, ethylenediaminetetraacetic acid (EDTA) was introduced to the electrolyte solution. This chelating ligand is known to coordinate cobalt ions, thus suppressing their OER activity.^[^ ^20^, ^43^ ^]^ Accordingly, after performing cyclic voltammetry (CV) in 0.1 m KOH in the OER potential range, EDTA (0.1 m aqueous solution) was added to the electrolyte (coded as EDTA + OER tested TpBpy‐Co in Figure 2b). This addition resulted in a minor decrease in current density (ca. 15% at +1.8 V vs RHE), indicating that free cobalt ions in the electrolyte do not act as the dominant active species of TpBpy‐Co. In contrast, exposing pristine TpBpy‐Co catalyst to EDTA‐containing KOH solution prior to the first OER polarization (coded as EDTA + pristine TpBpy‐Co) decreased the OER activity by almost three orders of magnitude, yielding a current density similar to that of an unmodified GCE. Determination of the amount of cobalt in the EDTA‐treated TpBpy‐Co powder and EDTA solution after COF‐exposure indicates that cobalt ions coordinated to TpBpy‐Co can be removed by EDTA and form an EDTA‐Co complex in the electrolyte (details shown in Supporting Information). This pair of EDTA treatment experiments demonstrates that while free cobalt ions in the electrolyte can be ruled out as the *dominant* active catalyst, the presence of cobalt ions in TpBpy‐Co is crucial for the development of OER activity in KOH(aq). To verify whether Co(II) ions leach out of the COF and form nanoparticles, and if so, what type of Co species are obtained, scanning electron microscopy (SEM) coupled to energy‐dispersive X‐ray analysis (EDX) was used to investigate the TpBpy‐Co morphology after exposure to OER conditions (1 min chronoamperometry at +1.8 V vs RHE). In neutral NaPB electrolyte, a homogeneous distribution of cobalt across the COF particles is observed, indicating structural integrity (see Figure S10, Supporting Information), despite the lack of OER activity. In contrast, during the same test in KOH(aq), TpBpy‐Co showed remarkable OER currents (see above), and the SEM‐EDX analysis revealed nanoparticles with increased cobalt and oxygen content (wt.%), as shown in Figure S11 (Supporting Information). This comparison indicates that transformation of the pre‐catalyst TpBpy‐Co to cobalt and oxygen‐based nanoparticle catalysts yields the OER active species in KOH, contradicting previous reports on the stability of this Co‐COF catalysts under OER conditions. Indeed since several cobalt and oxygen based compounds, such as Co(OH)_2_, CoOOH, and Co_3_O_4_, have been reported as highly active OER catalysts,^[^ ^44^, ^45^ ^]^ identification of the mechanism of particle formation and the compound species is performed next.

Indeed, nanoparticle formation can originate from a weakened coordination of Co(II) ions to the bipyridine units in alkaline electrolyte, the application of positive potentials in alkaline media, or a synergy of both. To distinguish between both, pristine TpBpy‐Co was immersed in 0.1 m KOH for 1 min under open circuit conditions, prior to SEM‐EDX analysis. As visible in Figure S12 (Supporting Information), cobalt based nanoparticles are formed spontaneously upon this immersion of the COF in the alkaline solution. In contrast, no nanoparticle formation is observed upon immersion in neutral electrolyte, maintaining homogeneous Co distribution among the COF particle, as shown in Figures S13 and S14 (Supporting Information). Moreover, we compared the PXRD patterns of the electrolyte immersed TpBpy‐Co samples with the pristine one (Figure 2c; Figure S15, Supporting Information). No new peaks are observed for the sample soaked in neutral electrolyte, while additional peaks corresponding to β‐Co(OH)_2_ emerge for the sample immersed in 0.1 m KOH for 1 min, in line with the SEM‐EDX results. The formation of β‐Co(OH)_2_ coincides with the observation of two redox transformations at ≈+1.2  and ≈+1.45 V versus RHE, respectively, which can be correlated to the conversion of β‐Co(OH)_2_ to CoOOH and of CoOOH to CoO_2_ (Figure 2d).^[^ ^44^, ^46^ ^]^ This observed conversion of cobalt(II) ions to cobalt hydroxide in alkaline media is thermodynamically favored as indicated in the Pourbaix diagram of Co (Figure S16, Supporting Information).^[^ ^47^, ^48^ ^]^ Of note, since PXRD only shows the crystalline components, the formation of other semicrystalline or amorphous cobalt hydroxide species besides β‐Co(OH)_2_ cannot be excluded.

**Figure 3:**
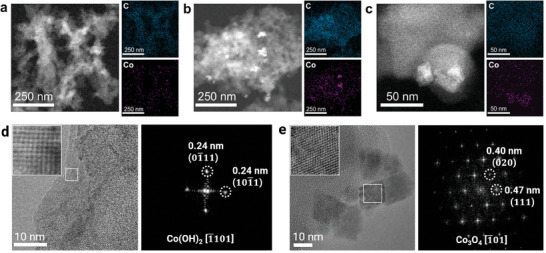
STEM images of the pristine, KOH exposed, and OER tested TpBpy‐Co. (a–c) High angle annular dark field (HAADF)‐STEM images and the corresponding EDX elemental mapping (carbon, cobalt) of pristine TpBpy‐Co (a), KOH exposed TpBpy‐Co (b), and OER tested TpBpy‐Co (c). (d,e) High resolution bright field (BF)‐STEM images with corresponding FFT patterns (labelled with crystallographic indices) from the boxed regions of KOH exposed TpBpy‐Co (d) and OER tested TpBpy‐Co (e).

To further understand the effects from electrolyte immersion and OER operation, post‐electrolysis characterization was carried out, including scanning transmission electron microscopy (STEM) and X‐ray photoelectron spectroscopy (XPS), and electron energy loss spectroscopy (EELS). Three different samples were compared, including pristine TpBpy‐Co, TpBpy‐Co immersed in 0.1 m KOH for 1 min (abbreviated as KOH exposed TpBpy‐Co), and TpBpy‐Co after 1 min OER test at +1.8 V versus RHE in 0.1 m KOH (abbreviated as OER tested TpBpy‐Co). For the preparation of KOH exposed and OER tested TpBpy‐Co samples, the pristine TpBpy‐Co was first drop‐casted onto a TEM grid (for STEM and EELS analysis) or a GCE (for XPS), and subsequently, exposure to KOH electrolyte at open circuit or OER potentials was performed. This enables direct STEM or XPS measurements for the samples on the corresponding substrates without further transfer. Pristine TpBpy‐Co was analyzed on the same type of substrate to ensure the validity of the comparison. As shown in the EDX elemental maps (**Figure** **3**a–c), pristine TpBpy‐Co shows overlapping carbon and cobalt signals at the scale of hundreds of nanometers, demonstrating homogeneous loading of Co(II) ions on TpBpy. In contrast, nanoparticles with brighter color are seen in the STEM images of both KOH exposed and OER tested TpBpy‐Co samples. Multivariate statistical analysis^[^ ^49^ ^]^ on the corresponding EDX spectrum imaging clearly indicates that the nanoparticles contain a higher amount of Co. The results are consistent with the above‐mentioned SEM‐EDX images and further confirm the formation of cobalt‐based nanoparticles. High‐resolution bright field (BF)‐STEM images and their corresponding Fast‐Fourier‐Transform (FFT) patterns reveal crystallographic information about these particles. KOH exposed TpBpy‐Co show crystalline nanoparticles with lattice spacing of 0.24 nm (Figure 3d), matching well with the $0\bar{1}11$ and $10\bar{1}1$ planes of β‐Co(OH)_2_, respectively, and suggesting a projection along the [$\bar{1}101$] zone axis. This is in accordance with the PXRD pattern of KOH exposed TpBpy‐Co, further proving the formation of β‐Co(OH)_2_ by a simple immersion in 0.1 m KOH. After OER operation in 0.1 m KOH, the nanoparticles formed show lattice spacings of 0.47 and 0.40 nm, corresponding to the 111 and 020 planes, respectively (Figure 3e). In fact, all spots can be indexed along the [$\bar{1}01$] zone axis of the mixed‐valence spinel Co_3_O_4_, suggesting that spinel Co_3_O_4_ nanoparticles are formed after subjecting the sample to OER conditions. Note that while spinel Co_3_O_4_ nanoparticles are found in the OER tested TpBpy‐Co, presumably not all cobalt species are transformed to spinel Co_3_O_4_, yet other amorphous or crystalline cobalt compounds and pristine TpBpy‐Co may remain alongside the spinel oxide after OER catalysis. As the Co_3_O_4_ phase is not detected in the PXRD patterns of the KOH exposed sample prior to OER studies, these studies demonstrate that Co_3_O_4_ formation requires positive potential to drive the transformation of Co(II) to the Co(II, III) species. This is in agreement with several reports studying cobalt and cobalt (oxy)hydroxides as OER catalysts, revealing that the real active phase is obtained via an in‐situ/operando structural transformation and (partial) Co valence change.^[^ ^44^, ^50^, ^51^, ^52^ ^]^

**Figure 4:**
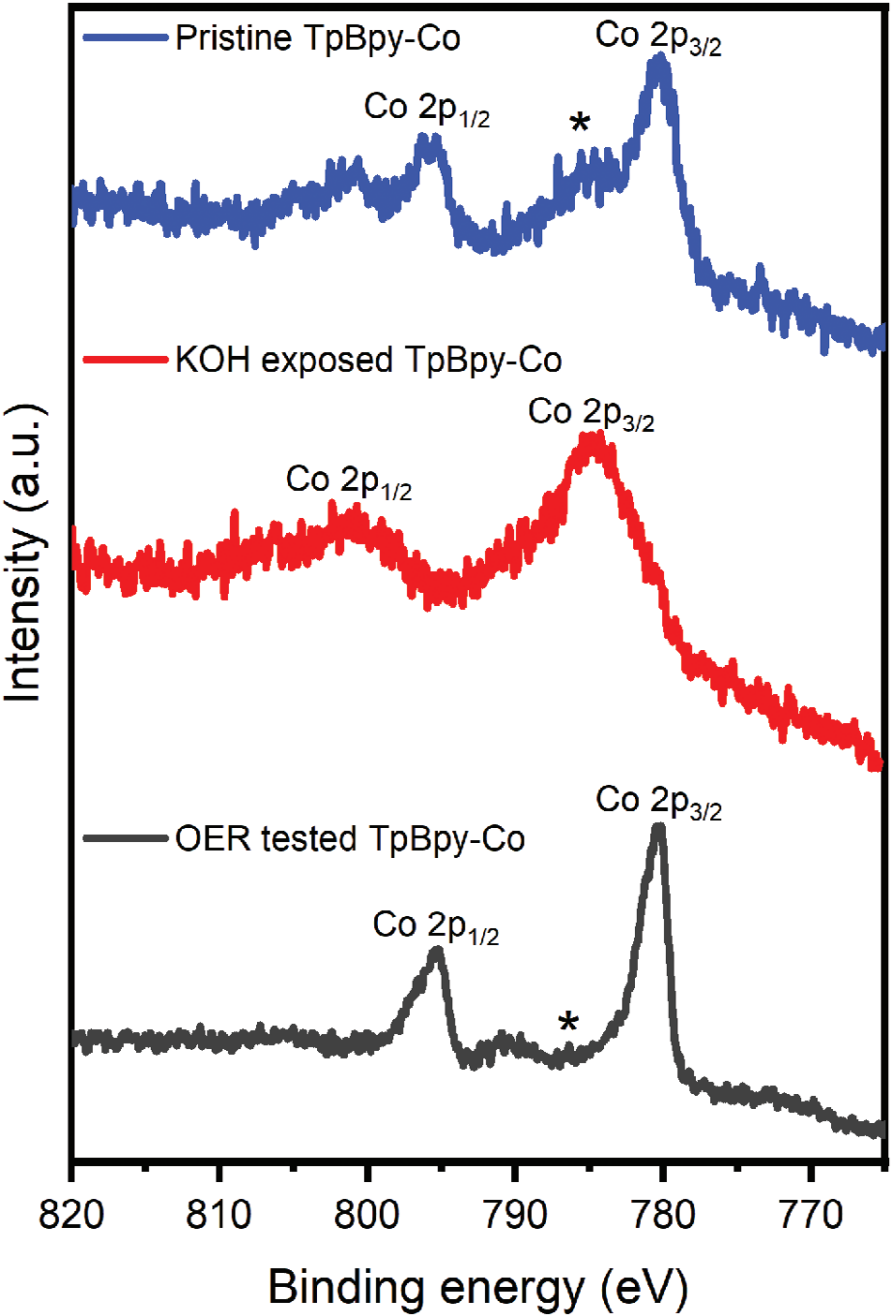
Co 2p XPS spectra of the pristine, KOH exposed and OER tested TpBpy‐Co. Asterisks indicate the satellite peak position of Co 2p_3/2_.

In addition, XPS analysis provides a comparison of the cobalt electronic environment among these three samples (**Figure** **4**). The pristine TpBpy‐Co exhibits two main peaks at 779.5  and 795.1 eV, corresponding to the 2p_3/2_ and 2p_1/2_ core levels of Co(II) ions, respectively. It is noticed that both 2p_3/2_ and 2p_1/2_ present considerably intense satellite peaks due to the presence of paramagnetic cobalt(II) ions, which has been observed for Co(II) molecular complexes and Co(II) ions contained in COFs.^[^ ^53^, ^54^, ^55^ ^]^ These pronounced satellite peaks are consistent with a cobalt oxidation state of +2 in pristine TpBpy‐Co. In comparison, exposing TpBpy‐Co to 0.1 m KOH produces a positive shift of 2p_3/2_ and 2p_1/2_ binding energies and significantly broadened peaks. Likely, this originates from the presence of multiple cobalt species with various ligand environments, such as β‐Co(OH)_2_, amorphous Co(OH)_2_ and some retained pristine TpBpy‐Co, existing in the sample after 1 minute of immersion in KOH. The OER tested TpBpy‐Co sample displays more structured 2p_3/2_ and 2p_1/2_ peaks at binding energies of 780.8  and 795.9 eV, respectively. Notably, the satellite peaks are less pronounced compared to pristine TpBpy‐Co, and closer to that of the Co_3_O_4_ Co 2p spectrum. The weakened satellite peaks typically imply a transformation of Co(II) to Co(III),^[^ ^56^, ^57^ ^]^ in agreement with the STEM results in Figure 3d,e. Besides, XPS fitting results of OER tested TpBpy‐Co also indicate the presence of Co(III), as shown in Figure S17 (Supporting Information). The transition from Co(II) to Co(III) is also associated with a shift toward lower binding energy, due to the combined effects of initial state charges, Madelung potentials, and final state relaxation energies.^[^ ^58^, ^59^ ^]^ The change in valence states of individual Co‐containing particles is further confirmed by STEM‐EELS. As shown in Figure S18 (Supporting Information), the Co‐L_3_ and L_2_ edges shift toward lower energy loss after OER operation, which is consistent with the shift in XPS Co 2p binding energy and the partial oxidation of Co(II) in Co(OH)_2_ to Co(III) in mixed‐valence Co_3_O_4_.

**Figure 5:**
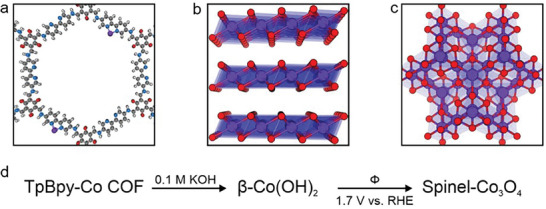
Schematic of the TpBpy‐Co transformation upon exposure to alkaline media and OER conditions. (a‐c) Structural model of TpBpy‐Co (a) and crystal structures of β‐Co(OH)_2_ (b) and spinel Co_3_O_4_ (c). (d) Co(II) ion transformation under alkaline electrolyte exposure and OER operational conditions.

The aforementioned characterizations reveal that the coordination between bipyridine and Co(II) ions is not strong enough in 0.1 m KOH electrolyte to suppress the spontaneous formation of Co based nanoparticles already after 1 min of exposure to alkaline conditions (**Figure** **5**). PXRD patterns, HR‐STEM images, and XPS of KOH exposed TpBpy‐Co demonstrate the formation of β‐Co(OH)_2_, which is known to be a very active OER catalyst. We suppose that other amorphous (hydrous) cobalt hydroxide species, potentially active for OER as well, co‐exist in the sample as the transformation of Co(II) ions occurs under mild conditions and Co speciation likely involves amorphous and semicrystalline (partially) hydrated phases. Upon applying a positive potential, Co(II) species are gradually oxidized to higher valence Co species, including spinel Co_3_O_4_, as seen in the HR‐STEM image of OER tested TpBpy‐Co.

The findings of our study suggest that structural integrity of metal ion‐modified COFs may not be taken for granted solely based on the observation of stable, high OER performances. Instead, a robust coordination between COF and metal ions is required to maintain the initial COF OER catalyst species. Indeed, the wide variety of COF building blocks, including those with a higher binding affinity to Co than Bpy, opens up the possibility of obtaining COFs with enhanced metal ion‐COF interactions. For example, COF‐366‐Co retains cobalt ions under the treatment of EDTA/KOH solution (0.1 m Na_4_EDTA in 0.1 m KOH solution) as demonstrated in Figure S19 (Supporting Information) and the experimental section (Supporting Information).

It is also worth noting that our results cannot fully eliminate the possibility that TpBpy‐Co is a secondary OER catalyst, albeit being chemically unstable. Further distinguishing the OER activity of TpBpy‐Co from the in‐situ formed cobalt based nanoparticles is experimentally impractical, since the COF and the inorganic Co‐based particles are intimately mixed, while the latter are very active OER catalysts. Therefore, we shift our focus to shedding light on whether coordinating Co(II) ions in TpBpy COF offers any distinctive catalytic advantage or disadvantage compared to free Co(II) ions. A control sample was prepared by physically mixing TpBz COF (where benzidine was used as the linker instead of bipyridine) with Co(OAc)_2_, and keeping the same cobalt amount (11 wt.%) compared to TpBpy‐Co (coded as TpBz+Co(OAc)_2_). In this case, the Co(II) ions in TpBz+Co(OAc)_2_ remain as free ions, as there is no distinct coordination interaction between Co(II) ions and TpBz. SEM‐EDX mapping reveals the appearance of particles with increased Co and O after 1 min immersion of TpBz+Co(OAc)_2_ in 0.1 m KOH compared with pristine TpBz+Co(OAc)_2_ (Figures S20 and S21, Supporting Information), similar to the case of TpBpy‐Co. In addition, the Co(II) ions are transformed to Co(OH)_2_ proven by STEM imaging (Figure S22d, Supporting Information), which is a common procedure to obtain amorphous Co(OH)_2_.^[^ ^47^ ^]^ Linear sweep voltammetry (LSV) curves with three replications of TpBpy‐Co and TpBz+Co(OAc)_2_ for electrochemical OER indicate that TpBz+Co(OAc)_2_ and TpBpy‐Co show a similar overpotential for OER (Figure S23, Supporting Information), which indeed suggests that TpBpy‐Co is unlikely to be more active than Co(OH)_2_ even if it works as a secondary OER catalyst. Moreover, high resolution STEM images of the post‐OER TpBz+Co(OAc)_2_ sample also indicate the appearance of spinel Co_3_O_4_, consistent with the observation in the post‐OER TpBpy‐Co (Figure S22e, Supporting Information).

Nevertheless, the PXRD of KOH exposed TpBz+Co(OAc)_2_ does not reveal the presence of β‐Co(OH)_2_ (Figure S24, Supporting Information), implying that the majority of the formed Co(OH)_2_ is amorphous and suggesting that the coordination of Co(II) ions on TpBpy‐COF facilitates, i.e., “seeds” the growth of crystalline β‐Co(OH)_2_. The above results demonstrate that while TpBpy‐Co does not provide a superior OER catalytic performance, the specific Co‐coordination interaction afforded by the bipyridine building blocks in TpBpy COF directs the growth of crystalline β‐Co(OH)_2_ nanoparticles. More generally, this “templating” or “scaffolding” role of the COF building blocks could potentially be exploited for the directed and controlled growth of the catalytically active species, both in terms of phase selectivity and morphology.

## 3 Summary and Conclusion

In summary, we have investigated the active species in the electrochemical OER catalyst TpBpy‐Co, which challenges the existing notion that an individual Co‐bpy center is the active species under alkaline reaction conditions. Specifically, we observe that TpBpy‐Co exhibits catalytic OER activity in 0.1 m KOH, but not in pH 7 buffer, which is attributed to the formation of cobalt hydroxide nanoparticles after soaking TpBpy‐Co in 0.1 m KOH for times as short as 1 min. The nature of the nanoparticles has further been confirmed by structural analysis via PXRD and HR‐STEM of KOH‐exposed TpBpy‐Co, revealing the formation of crystalline cobalt hydroxide (β‐Co(OH)_2_). After OER operation, spinel Co_3_O_4_ nanoparticles are found by STEM post‐electrolysis characterization, indicating the transformation of Co(II) to Co(III) upon applying a positive potential of +1.8 V versus RHE. Our findings suggest that the primary active species of TpBpy‐Co in 0.1 m KOH are cobalt oxide‐based nanoparticles, rather than Co ions coordinated to the COF Bpy moieties as previously assumed. Our study further reveals that although TpBpy‐Co does not act as the primary active species, the specific coordination between Co(II) ions and TpBpy COF directs the formation of crystalline β‐Co(OH)_2_, while the formation of amorphous cobalt hydroxide is observed when using benzidine linkers without specific metal coordination sites.

Our study suggests that in the absence of a strong coordination between a metal center and COF, the true active species may evolve under *operando* conditions into a “classical” heterogeneous catalyst which is distinct from the idealized metal coordination complex often invoked to be the COF active site. In other words, engineering strong and specific bonding between the COF linker and the metal catalyst is crucial to achieve COF electrocatalysts with well‐defined, heterogenized metal complex sites as the actual catalytically active species. While recent years have witnessed an accelerating development of employing COFs as electrocatalysts, efforts directed at identifying and controlling the nature of the active species are much less developed. The results presented here highlight that catalytic activity is only the first step in the rational design of COF electrocatalysts, which need to be complemented by studies identifying the “true” active species to evaluate the suitability of COF candidate structures for electrocatalysis. While our study reveals that TpBpy‐Co may not show inherent OER activity due to its instability under OER working conditions, the rational choice of COF building blocks presents a versatile platform to design robust COF electrocatalysts, given the wealth of structurally diverse covalent organic framework compounds.

## Conflict of Interest

The authors declare no conflict of interest.

## Author Contributions

P.H. and A.R.C. contributed equally to this work. P.H. performed the electrochemical experiments and post‐electrolysis characterization. A.R.C. performed the syntheses and characterization of the COFs, as well as some control experiments. Y.J., S.Z. and C.S. performed STEM, EDS, and EELS characterization and analysis. L.Y., B.V.L., and K.T. conceived and supervised the research and discussed the data. All authors co‐wrote, read and commented on the manuscript.

## Supporting information



Supporting Information

## Data Availability

The data that support the findings of this study are available from the corresponding author upon reasonable request.
