# Supplementary material for: Shedding Light on the Active Species in a Cobalt‐Based Covalent Organic Framework for the Electrochemical Oxygen Evolution Reaction
Source: Adv Sci (Weinh). 2024 Nov 26;12(3):2413555. doi: 10.1002/advs.202413555 (PMC11744715; doi:10.1002/advs.202413555)
Supplement: Supplementary file 1 — Supporting Information [file ADVS-12-2413555-s001.docx]

Supporting Information

Shedding Light on the Active Species in a Cobalt-Based Covalent Organic Framework for the Electrochemical Oxygen Evolution Reaction

Pouya Hosseini^1,3+^, Andrés Rodríguez-Camargo^2,4+^, Yiqun Jiang^3^, Siyuan Zhang^3^, Christina Scheu^3^, Liang Yao^2,5*^, Bettina V. Lotsch^2,4,6*^, and Kristina Tschulik^1,3*^

[1] P. Hosseini, K. Tschulik

Faculty of Chemistry and Biochemistry Analytical Chemistry II, Ruhr-Universität Bochum, Universitätsstrasse150, 44801 Bochum, Germany
E-mail: [kristina.tschulik@rub.de](mailto:kristina.tschulik@rub.de)

[2] A. Rodríguez-Camargo, L. Yao, B. V. Lotsch
Nanochemistry Department, Max Planck Institute for Solid State Research

Heisenbergstraße 1, 70569 Stuttgart, Germany

E-mail: [b.lotsch@fkf.mpg.de](mailto:b.lotsch@fkf.mpg.de)

[3] P. Hosseini, Y. Jiang, S. Zhang, C. Scheu, K. Tschulik

Max Planck Institute for Sustainable Materials Max-Planck-Straße 1, 40237 Düsseldorf, Germany

[4] A. Rodríguez-Camargo, B. V. Lotsch

Department of Chemistry, University of Stuttgart, Pfaffenwaldring 55, 70569 Stuttgart, Germany

[5] L. Yao

State Key Laboratory of Luminescent Materials and Devices, Institute of Polymer Optoelectronic Materials and Devices, Guangdong Basic Research Center of Excellence for Energy and Information Polymer Materials, South China University of Technology, Guangdong 510640, China

Email: [liangyao@scut.edu.cn](mailto:liangyao@scut.edu.cn)

[6] B. V. Lotsch

Department of Chemistry, University of Munich (LMU), Butenandtstraße 5–13, 81377 München, Germany

+These authors contributed equally to this work.

**Table of Content**

[Chemicals and materials 4](#_Toc178590954)

[Instruments 4](#_Toc178590955)

[Synthetic procedures 5](#_Toc178590956)

[Electrochemistry 6](#_Toc178590957)

[EDTA treatment experiments 7](#_Toc178590958)

[Figure S1. Schematic representation of the electrochemical cell. 8](#_Toc178590959)

[Figure S2. Kubelka-Munk function, normalized F(R), plotted against wavelength converted from the diffuse reflectance data. Inset graph shows the photographs of TpBpy (left) and TpBpy-Co (right) powders. 9](#_Toc178590960)

[Figure S3. Tauc plots of TpBpy and TpBpy-Co COFs and extracted optical band gaps. 9](#_Toc178590961)

[Figure S4. Structure model of TpBpy. (a) Simulated TpBpy structure with AA stacking mode. (b) Indexed PXRD pattern (Cu-K_α1_) with corresponding Pawley refinement (cyan) and its R-factors (R_wp_ for weighted residual factor and R_p_ for residual of least-squares refinement). (c) Unit cell parameters of refined TpBpy structure model. 10](#_Toc178590962)

[Figure S5. BET plot of TpBpy showing the relative pressure (P/P_0_) vs. 1/[W((P_0_/P)-1)] BET function. 11](#_Toc178590963)

[Figure S6. BET plot of TpBpy-Co showing the relative pressure (P/P_0_) vs. 1/[W((P_0_/P)-1)] BET function. 11](#_Toc178590964)

[Figure S7. Cumulative pore volume and pore size distribution of TpBpy. 12](#_Toc178590965)

[Figure S8. Cumulative pore volume and pore size distribution of TpBpy-Co. 12](#_Toc178590966)

[Figure S9. (a) Normalized experimental LSVs collected from TpBpy-Co in 0.1 M KOH (pH 12.9), at a scan rate of 10 mV s^–1^ in black. Normalized first and second derivatives are reported in blue and red, respectively. (b) Tafel plot of TpBpy-Co. 13](#_Toc178590967)

[Figure S10. SEM-EDX elemental analysis of TpBpy-Co on GCE after 1 min OER in 0.5 M NaPB pH 7. Scale bar 2.5 μm. 14](#_Toc178590968)

[Figure S11. SEM-EDX elemental analysis of TpBpy-Co on GCE after 1 min OER in 0.1 M KOH pH 12.9. Scale bar 2.5 μm. 15](#_Toc178590969)

[Figure S12. SEM-EDX elemental mapping of TpBpy-Co after 1-minute immersion in 0.1 M KOH pH 12.9, on an ITO substrate. Scale bar 2.0 μm. 16](#_Toc178590970)

[Figure S13. SEM-EDX elemental mapping of pristine TpBpy-Co on an ITO substrate. Scale bar 2.0 μm. 17](#_Toc178590971)

[Figure S14. SEM-EDX elemental mapping of TpBpy-Co after 1-minute immersion in 0.5 M NaPB pH 7, on an ITO substrate. Scale bar 5.0 μm. 18](#_Toc178590972)

[Figure S15. PXRD pattern comparison of KOH exposed TpBpy-Co and simulated Co(OH)_2_, CoOOH and Co_3_O_4_. 19](#_Toc178590973)

[Figure S16. Pourbaix diagram of cobalt species in an aqueous medium at 25 °C.^[8]^ 20](#_Toc178590974)

[Figure S17. X-ray photoelectron spectra of Co 2p of pristine TpBpy-Co and OER treated TpBpy-Co and peak fitting of Co 2p_3/2_ spectrum (open circles: experimental data, dark line: envelop and light grey line: background). 21](#_Toc178590975)

[Table S1. Co 2p_3/2_ spectral fitting parameters (BE.: Binding energy, L.Sh.: line shape and FWHM: full width at half-maximum). 22](#_Toc178590976)

[Figure S18. EELS spectra (a) collected from the area within the green box of KOH exposed TpBpy-Co (b) and OER tested TpBpy-Co (c). 23](#_Toc178590977)

[Figure S19. UV-Vis absorption spectra of the EDTA/KOH solution (0.1 M Na_4_EDTA in 0.1 M KOH solution) after immersing Co(OAc)_2_, TpBpy-Co or COF-366-Co in the solution for 1 min. It can be seen that the exposure of Co(OAc)_2_ and TpBpy-Co to EDTA/KOH solution leads to the appearance of Co-EDTA complex absorption around 550 nm. In comparison, COF-366-Co does not show noticeable Co-EDTA complex absorption after immersing in EDTA/KOH solution. The results suggest that COF-366-Co coordinates the Co(II) ion more strongly. 24](#_Toc178590978)

[Figure S20. SEM-EDX elemental mapping of pristine TpBz+Co(OAc)_2_ on an ITO substrate. Scale bar 10.0 μm. 25](#_Toc178590979)

[Figure S21. SEM-EDX elemental mapping of pristine TpBz+Co(OAc)_2_ after 1-minute immersion in 0.1 M KOH pH 12.9, on an ITO substrate. Scale bar 10.0 μm. 26](#_Toc178590980)

[Figure S22. STEM images of the pristine, KOH exposed, and OER tested TpBz+Co(OAc)_2_. (a-c) High angle annular dark field (HAADF)-STEM images and the corresponding EDX elemental mapping (carbon, cobalt) of pristine TpBz+Co(OAc)_2_ (a), KOH exposed TpBz+Co(OAc)_2_ (b), and OER tested TpBz+Co(OAc)_2_ (c). (d, e) High resolution bright field (BF)-STEM images with corresponding FFT patterns (labelled with crystallographic indices) from the boxed regions of KOH exposed TpBz+Co(OAc)_2_ (d) and OER tested TpBz+Co(OAc)_2_ (e). 27](#_Toc178590981)

[Figure S23. Current density (geometric) – potential curves of TpBpy-Co, TpBz+Co(OAc)_2_ and GCE in 0.1 M KOH with 10 mV s^–1^ scan rate. 28](#_Toc178590982)

[Figure S24. Experimental PXRD pattern of KOH exposed TpBz + Co(OAc)_2_ and simulated diffraction pattern of β-Co(OH)_2_. 28](#_Toc178590983)

[References 29](#_Toc178590984)

# Chemicals and materials

All chemicals were purchased from commercial suppliers without any further purification: [2,2´-bipyridine]-5,5´-diamine (Bpy, Biosynth, 98%), benzidine (Bz, Sigma-Aldrich, 98%), hexamethylenetetramine (Alfa Aesar, 99+%), anhydrous phloroglucinol (Acros organics, 99+%), trifluoroacetic acid (TFA, Carl-Roth, 100%), acetic acid (AcOH, Carl-Roth, 100%), 1,4-dioxane (extra dry, Acros organics, 99.5%), N,N-dimethylacetamide (DMAc, extra dry, Acros organics, 99.8%), 1,2-dichlorobenzene (o-DCB, extra dry, Acros organics, 98+%), pyrrolidine (Py, Sigma-Aldrich, 99%), methanol (MeOH, Carl-Roth, 99.9%), cobalt acetate tetrahydrate (Co(OAc)_2_· 4H_2_O, Alfa Aesar, 98%), dichloromethane (DCM, Carl-Roth, 99.5%), potassium hydroxide (KOH, Sigma-Aldrich semiconductor grade, pellets, 99.99% trace metals basis), Sodium phosphate dibasic dihydrate (NaH_2_PO_4_·2H_2_O, Sigma-Aldrich, 99%), Sodium phosphate monobasic monohydrate (Na_2_HPO_4_, Sigma-Aldrich, 99.95%), and ethylenediaminetetraacetic acid tetrasodium salt hydrate (Na_4_EDTA, Sigma-Aldrich, 99%) was used for the synthesis and electrochemical experiments.

# Instruments

Powder X-ray diffraction (PXRD): Powder X-ray diffraction were performed on a Stoe Stadi P diffractometer (Cu-Kα_1_, Ge(111) in Debye-Scherrer geometry, using sealed glass capillaries (*OD* = 0.7 mm).

Sorption: Nitrogen sorption isotherms were measured on an Autosorb iQ MP from Quantachrome Instruments at 77 K. Prior to gas adsorption experiments, samples were degassed for 12 h at 120 °C under vacuum. Pore size distributions were calculated using the Quenched Solid Density Functional Theory (QSDFT), in equilibrium mode on a carbon material with cylindrical pores as a model. For the multipoint BET surface area calculations, the range between 0.05 and 0.35 P/P_0_ was chosen, where a linear trend was observed in BET plots.

UV–Vis diffuse reflectance spectra (UV-Vis DRS): UV-Vis reflectance spectra were collected on a Cary 5000 spectrometer (referenced to barium sulphate). Subsequently, UV-Vis DRS spectra were calculated from the reflectance data using the Kubelka-Munk approach. The optical band gaps were estimated by Tauc plot assuming a direct transition.

**UV-Vis spectra of the solutions:** The UV-Vis absorption spectra was recorded in an Agilent Technologies Cary 60 spectrometer using a quartz cuvette (1 cm path length).

Liquid nuclear magnetic resonance (NMR): Liquid state ^1^H-NMR spectra was recorded on a JEOL ECZ 400S 400 MHz spectrometer and is reported as follows: chemical shift δ in ppm (multiplicity, number of protons, assignment). Couplings are expressed as: s (singlet). All chemical shifts δ are reported to the nearest 0.01 ppm with the residual solvent peak as the internal reference (chloroform-d = 7.26 ppm).

ICP-OES: ICP-OES measurements for cobalt amount determination were performed on a Varian Vista-PRO simultaneous spectrometer (Agilent Technologies, Santa Clara, California, USA) with a CCD-detector. Samples were treated by microwave digestion with Discover SP-D from CEM GmbH (sample dissolved in HNO_3_ (65%) at 185 °C for 25 min./diluted with double distilled water).

X-ray photoelectron spectroscopy (XPS): XPS measurements were carried out with an ultra-high vacuum (UHV) setup equipped with a monochromatic Al Kα X-ray source (1486.3 eV; anode operating at 14.5 kV and 30.5 mA) and a high-resolution Gammadata-Scienta SES 2002 analyzer. The spectra were recorded in the fixed transmission mode with a pass energy of 200 eV. Charging effects were compensated by applying a flood gun. Binding energies were calibrated by positioning the main C 1s peak at 284.8 eV. The samples for post-electrolysis XPS characterization were obtained by casting COF samples on glassy carbon electrode and treating with corresponding conditions.

The spectra were analyzed using CasaXPS software version 2.3.14, wherein Gaussian-Lorentzian (GL) profiles with a 70:30 ratio were employed for each component (GL (30)). A standard Shirley background was utilized for the spectra. All spectra were referenced to remaining adventitious carbon at 284.8 eV. To ensure precision, the peak area ratios and peak splitting values (Table S1) were constrained based on reference measurements.^[1]^

Scanning electron microscopy (SEM): SEM images were measured with a Zeiss Merlin using electron high tension voltage of 5 kV. Energy dispersive X-ray spectroscopy (EDX) mapping was carried out using the Oxford energy dispersive X-ray detector. The measurements of OER tested samples were performed directly on glassy carbon electrodes. Except those, samples were dispersed in isopropyl alcohol and drop casted onto indium tin oxide (ITO) substrates.

Scanning transmission electron microscopy (STEM): COF samples were drop-casted on gold grids. For post-electrolysis samples, electrochemical experiments were directly carried out on the gold TEM grids and then dried for imaging.

STEM measurements were performed on a probe-corrected JEOL 2200fs operated at 200 kV, as well as a probe-corrected Titan Themis operated at 300 kV. The aberration-corrected STEM probe has a convergence angle of 24 mrad. High angle annular dark field (HAADF) and bright field (BF)-STEM images were recorded using detectors with collection angles of 73-200 and 0-7 mrad, respectively. STEM-EDX spectrum imaging was performed using a SuperX detector. STEM-electron energy loss spectroscopy (EELS) spectrum imaging was recorded on a Gatan Quantum ERS spectrometer. Multivariate statistical analysis^[2]^ was performed on the spectrum imaging datasets to denoise the data and highlight the chemical segregation.

# Synthetic procedures

Triformylphloroglucinol (Tp)**:** Tp was synthesized based on a previous report.^[3]^ In a three-neck flask equipped with a condensing system, 15.1 g hexamethylenetetramine (108 mmol) and 6.0 g anhydrous phloroglucinol (49 mmol) were dissolved using 90 mL trifluoroacetic acid (TFA) under N_2_ atmosphere. The solution was then heated at 100 °C for 2.5 h. Subsequently, 150 mL of 3 M HCl was added and the reaction was maintained at 100 °C for 1 h more. After reaction time was completed, the reaction was cooled down to room temperature and the solids were removed by filtration on filter paper. Next, the acidic solution was extracted with DCM (*ca*. 100 mL 3 times) and the organic phase was dried under magnesium sulphate and filtered. After rotary evaporation of the organic phase, 1.24 g (12% yield) of pale-yellow powder was collected. ^1^H NMR (400 MHz, CDCl_3_): δ 14.10 (s, 3H, OH), 10.14 (s, 3H, CHO) ppm.

TpBpy COF synthesis: TpBpy COF was synthesized *via* Schiff-base condensation reaction, following a previous report.**^[4]^** In a 10 mL glass vial, 63.0 mg (0.3 mmol) of Tp and 83.7 mg (0.45 mmol) of Bpy were dispersed in 1.5 mL of o-dichlorobenzene (o-DCB) and 4.5 mL of dimethylacetamide (DMAc) after 10 min of sonication. Then, 0.6 mL of 6 M acetic acid were added as a catalyst. The color of the solution quickly changed to orange, as a sign that polymerization started immediately. The glass vial was sealed and heated at 120 °C for 3 days. After the reaction, the resulting COF powder was filtered and sequentially washed with DMAc, acetone and DCM. The orange TpBpy COF powder was dried in ambient air overnight.

TpBpy-Co COF synthesis: For cobalt acetate loading, 30 mg of TpBpy COF were immersed in a methanolic solution of Co(OAc)_2_ 4H_2_O (20 mg of Co(OAc)_2_ 4H_2_O in 20 mL of methanol) and stirred for 4 hours at room temperature.^[4]^ TpBpy-Co was obtained as red powder after washing with copious amount of methanol and dried in air.

TpBz COF synthesis: In this case, pyrrolidine was used as catalyst following a previous report.**^[5]^** 16.8 mg (0.08 mmol) of Tp and 22.3 mg (0.12 mmol) of Bpy were charged in a 10 mL glass vial, followed by adding 1 mL of 1,4-dioxane and 20 μL of pyrrolidine. The glass vial was sealed, sonicated for 10 min, and kept at 120 °C for 3 days. After the reaction, COF powder was filtered and washed with acetone, THF and DCM.

TpBz+Co(OAc)_2_ preparation: The sample was prepared by physically mixing an equal amount by mass of synthesized TpBz (20 mg) and Co(OAc)_2_·4H_2_O (20 mg). The two powders were mixed in an agate mortar after light grinding. Cobalt amount was determined to be 11 wt% by ICP-OES.

COF-366-Co synthesis: COF-366-Co was synthesized with the procedure developed by our group very recently.^[6]^

# Electrochemistry

Electrochemical cell and techniques

To avoid Fe contamination high purity KOH was used (semiconductor grade, pellets, 99.99% trace metals basis) for electrolyte preparation. In addition, all glassware was cleaned by immersion in 1 g/L KMnO_4_ solution acidified with 20 mL/L of 96% (m/m) H_2_SO_4_ for at least 24 h. Subsequently, glassware was immersed in a solution made of 40 mL/L 30% H_2_O_2_ acidified with 20 mL/L of 96% (m/m) H_2_SO_4_ until there was no visual evidence of purple color. Afterwards, the glassware was rinsed thoroughly with ultrapure water and boiled in ultrapure water for three times. Ultra-pure water obtained with a Thermo Scientific Barnstead Gen-Pure xCAD Plus Ultrapure Water Purification System (conductivity: 0.055 µS cm^−1^ at 25 ºC) was employed to prepare the electrolytes for electrochemical experiments.

Electrochemical behavior and OER activity of the materials were studied and measured by cyclic voltammetry (CV), linear sweep voltammetry (LSV) and chronoamperometry using a Metrohm Autolab potentiostat. The results have been repeated multiple times to confirm the validity of the data. A standard three-electrode configuration was used (Figure S1), composed of a reference electrode (RHE, HydroFlex® Hydrogen Reference Electrode), a counter electrode (graphite rod) and a working electrode (samples deposited on glassy carbon electrodes with a diameter of 5 mm) rotating at 1600 rpm. The reference electrode was located in a Luggin capillary to located the sensing point of the reference electrode close to the working electrode. The counter electrode was separated from the main compartment by a glass frit. LSVs were measured from 1.4 to 1.7 V vs RHE with a scan rate of 10 mV s^−1^ in 0.1 M KOH (pH 12.9) and 0.5 M NaPB (sodium phosphate buffer, pH 7.0). For Tafel analysis, 15 cyclic voltammetry (CV) cycles were performed within the voltage range of 1.4 to 1.7 V under Rotating Disk Electrode (RDE) conditions. Subsequently, a Linear Sweep Voltammetry (LSV) was carried out to determine the Tafel slope. The current density data were obtained by dividing the electrochemical current by the geometry area of the electrode.

EDTA treatment was performed by exposing the corresponding COF modified GCE electrode in 0.1 M Na_4_EDTA aqueous solution containing 0.1 M KOH for 1 min and subsequently rinsing with ultra-pure water to remove the residual solution prior to electrochemical investigations.

The Tafel analysis approach, developed by Corva et al.,^[7]^ utilizes derivatives of the electrochemical current as a benchmark for kinetic analysis. By analyzing these derivatives, it is possible to obtain straightforward and dependable information about the underlying reaction mechanisms. The exponential dependency of the derivatives is similar to that of the original current data, making it easier to identify which mechanisms are affecting the electrochemical currents, while constant background currents are removed. This approach was selected since it provides a more robust method for investigating the kinetics of electrochemical reactions than typical routes towards identifying and analyzing Tafel regions in LSV or CV curves.

All potentials presented here are Ohmic-drop corrected using the resistance value obtained from electrochemical impedance spectroscopy (EIS) measurements (Ru). EIS measurements were carried out using the same electrochemical system at open circuit potential (OCP) within a frequency range of 100’000 to 0.1 Hz of a sinusoidal perturbation of 10 mV amplitude.

Working electrode preparation: 2 mg COF was dispersed in 1 mL of an isopropanol : water mixture (v/v=3:2) by stirring for 12 hours at 500 rpm. 10 μL of the prepared COF ink were drop cast on a polished GCE with a diameter of 5 mm. The working electrode was dried in air for 12 hours before use.

# EDTA treatment experiments

ICP-OES measurement for quantifying cobalt amount: 10 mg of TpBpy-Co was stirred in 5 mL of 0.1 M Na_4_EDTA in 0.1 M KOH solution for 1 minute. Then, the COF powder and solution were separated by filtration. ICP-OES analysis was performed to determine the cobalt amount of the pristine TpBpy-Co, EDTA treated TpBpy-Co and TpBpy-Co exposed EDTA KOH solution, where the cobalt amount is found to be 11 wt%, 6 wt% and 98 mg/mL, respectively. Note that for EDTA treatment electrochemical measurement, only 0.02 mg of TpBpy-Co were deposited on the glassy carbon electrode and more EDTA KOH solution were used for treating the sample. Hence, it is expected that the remaining cobalt amount of TpBpy COF on glassy carbon electrode is smaller after EDTA treatment compared to this experiment.

UV-Vis spectra of the solution: For EDTA treatment, 5 mg of TpBpy-Co, COF-366-Co or Co(OAc)_2_·4H_2_O was stirred in 10 mL of 0.1 M Na_4_EDTA in 0.1 M KOH solution for 1 minute. The solution was then collected with a 3 mL syringe and passed through a 0.22 µm hydrophilic PTFE filter to remove the remaining powder. Subsequently, the solution was diluted with 0.1 M KOH solution to record a qualitative UV-Vis absorption spectrum of the Co-EDTA complex formed.


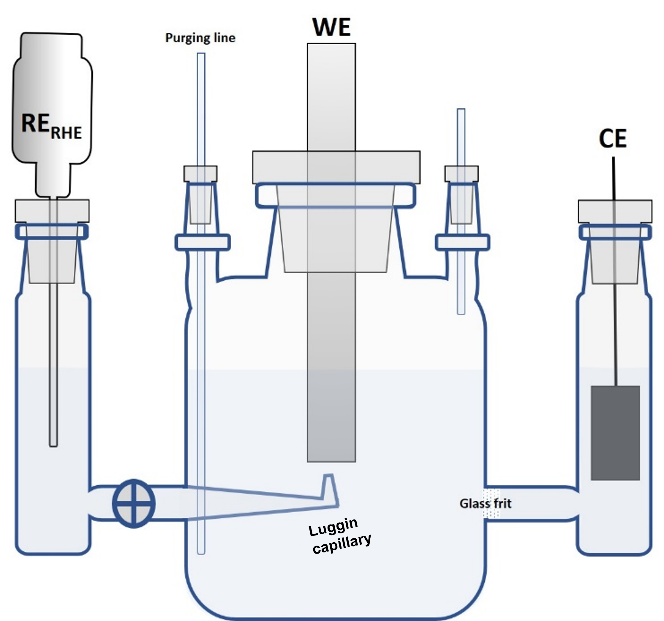


# Figure S1. Schematic representation of the electrochemical cell.

# Figure S2. Kubelka-Munk function, normalized F(R), plotted against wavelength converted from the diffuse reflectance data. Inset graph shows the photographs of TpBpy (left) and TpBpy-Co (right) powders.

# Figure S3. Tauc plots of TpBpy and TpBpy-Co COFs and extracted optical band gaps.


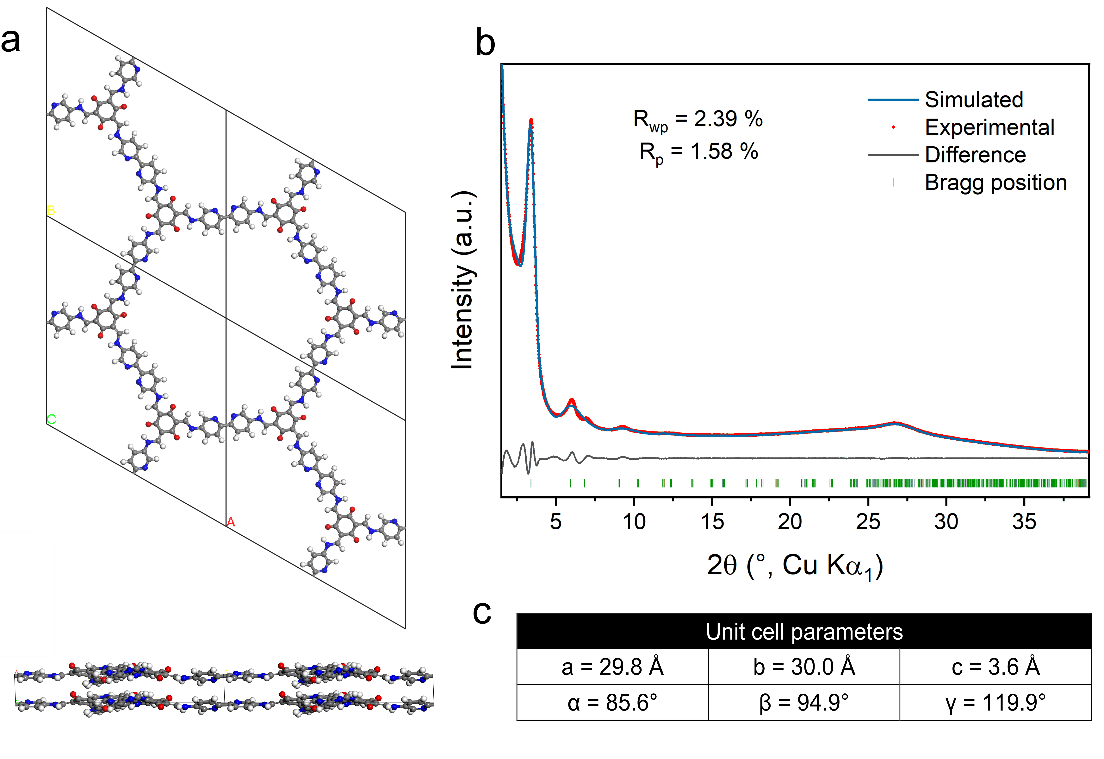


# **Figure S4.** Structure model of TpBpy. (a) Simulated TpBpy structure with AA stacking mode. (b) Indexed PXRD pattern (Cu-K_α1_) with corresponding Pawley refinement (cyan) and its R-factors (R_wp_ for weighted residual factor and R_p_ for residual of least-squares refinement). (c) Unit cell parameters of refined TpBpy structure model.

# Figure S5. BET plot of TpBpy showing the relative pressure (P/P_0_) vs. 1/[W((P_0_/P)-1)] BET function.

# Figure S6. BET plot of TpBpy-Co showing the relative pressure (P/P_0_) vs. 1/[W((P_0_/P)-1)] BET function.


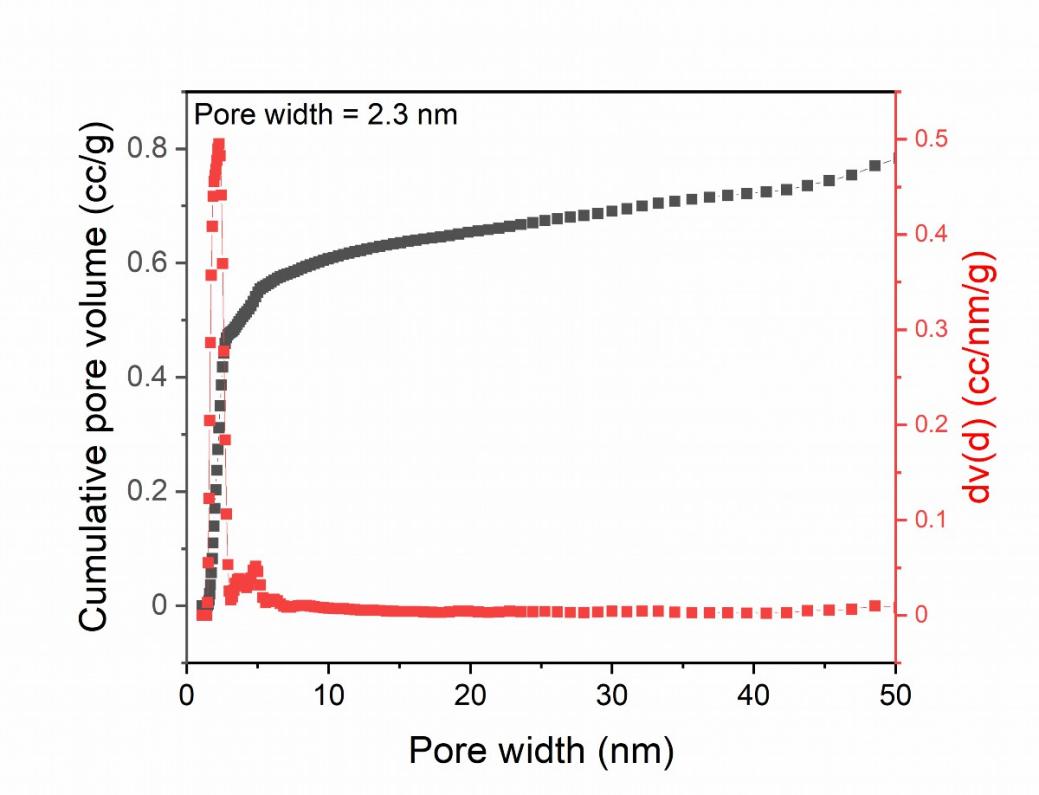


# Figure S7. Cumulative pore volume and pore size distribution of TpBpy.

**
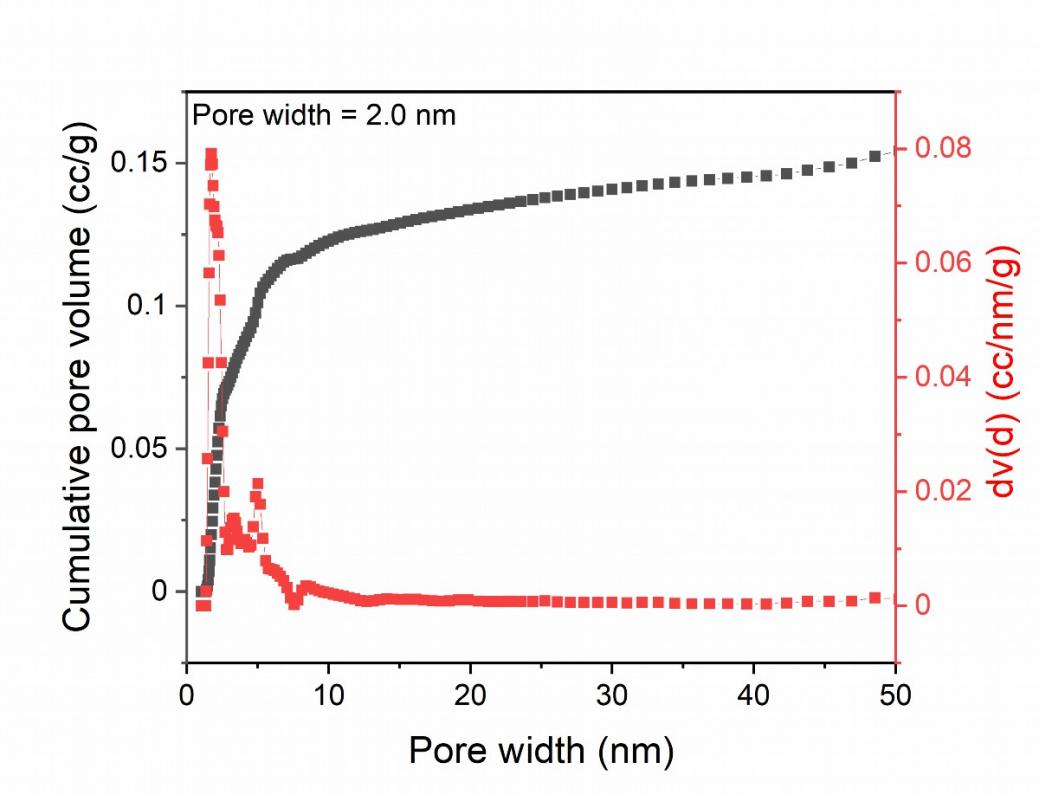
**

# Figure S8. Cumulative pore volume and pore size distribution of TpBpy-Co.


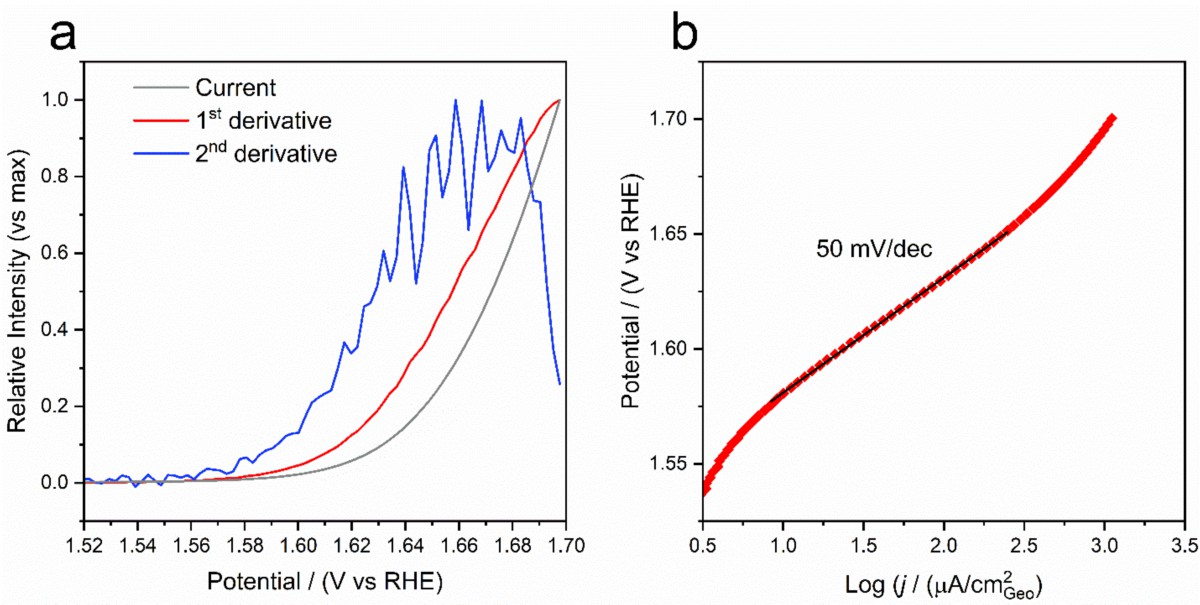


# **Figure S9.** (a) Normalized experimental LSVs collected from TpBpy-Co in 0.1 M KOH (pH 12.9), at a scan rate of 10 mV s^–1^ in black. Normalized first and second derivatives are reported in blue and red, respectively. (b) Tafel plot of TpBpy-Co.


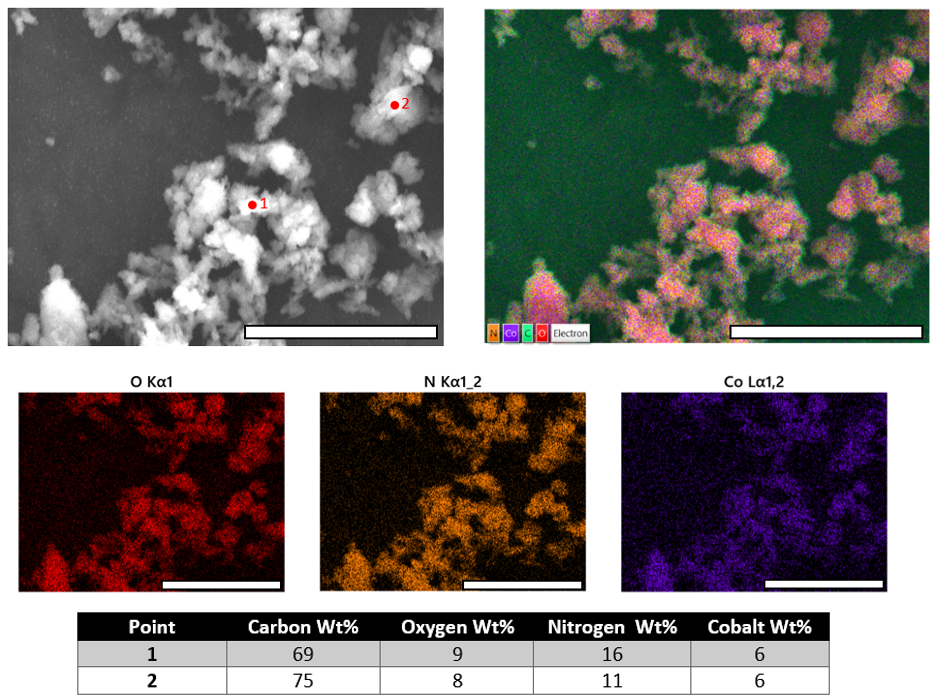


# Figure S10. SEM-EDX elemental analysis of TpBpy-Co on GCE after 1 min OER in 0.5 M NaPB pH 7. Scale bar 2.5 μm.


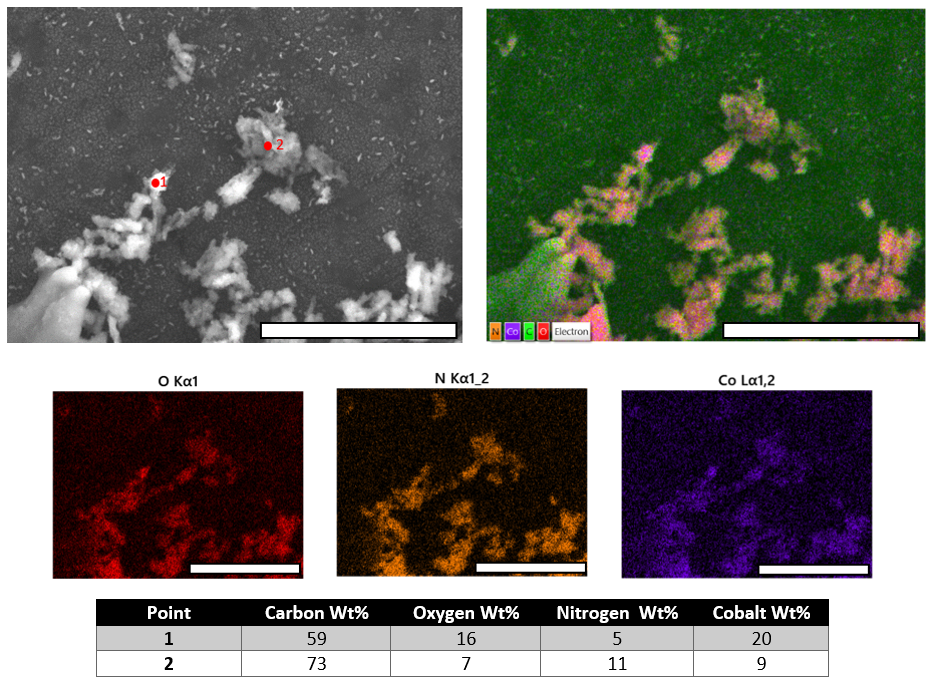


# Figure S11. SEM-EDX elemental analysis of TpBpy-Co on GCE after 1 min OER in 0.1 M KOH pH 12.9. Scale bar 2.5 μm.


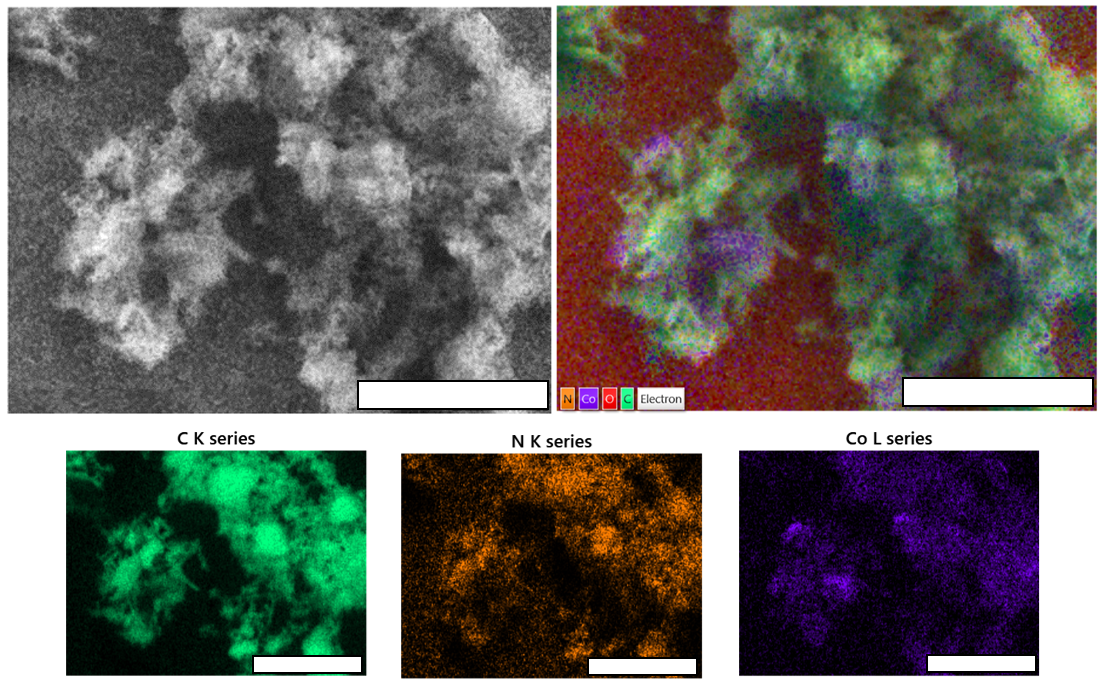


# Figure S12. SEM-EDX elemental mapping of TpBpy-Co after 1-minute immersion in 0.1 M KOH pH 12.9, on an ITO substrate. Scale bar 2.0 μm.


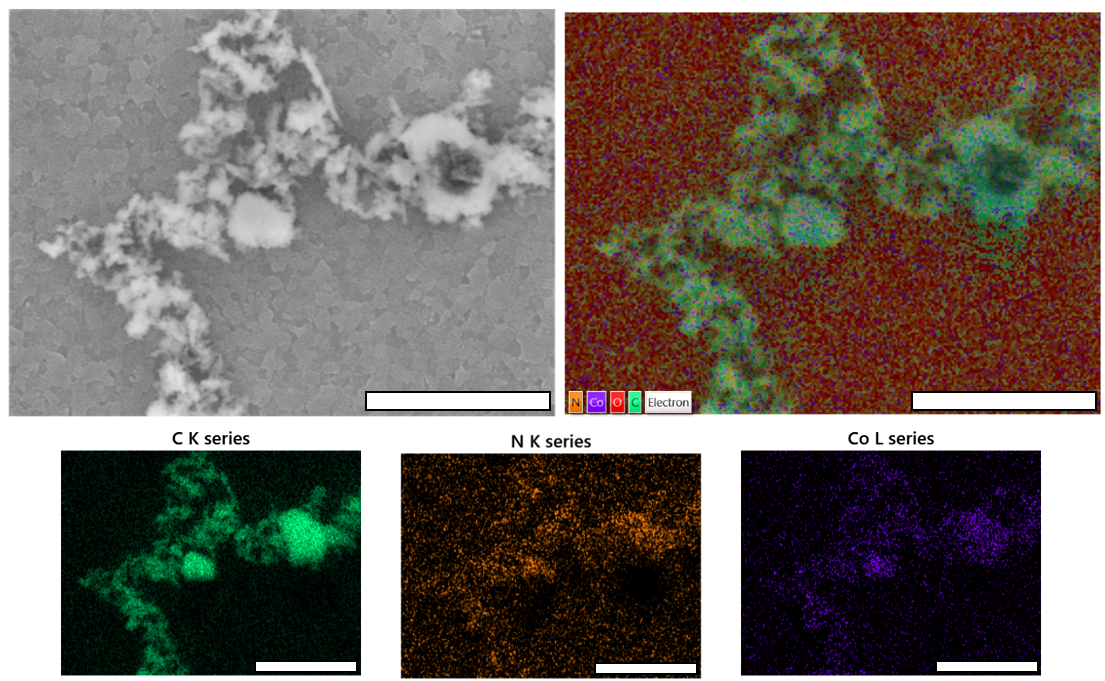


# Figure S13. SEM-EDX elemental mapping of pristine TpBpy-Co on an ITO substrate. Scale bar 2.0 μm.


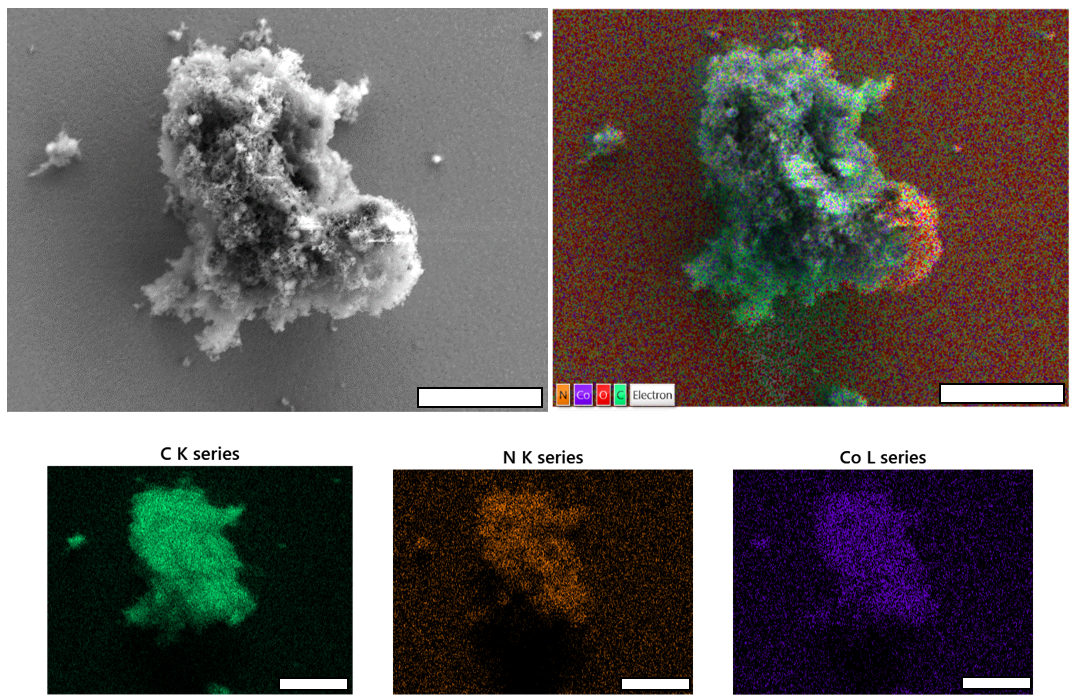


# Figure S14. SEM-EDX elemental mapping of TpBpy-Co after 1-minute immersion in 0.5 M NaPB pH 7, on an ITO substrate. Scale bar 5.0 μm.

# Figure S15. PXRD pattern comparison of KOH exposed TpBpy-Co and simulated Co(OH)_2_, CoOOH and Co_3_O_4_.


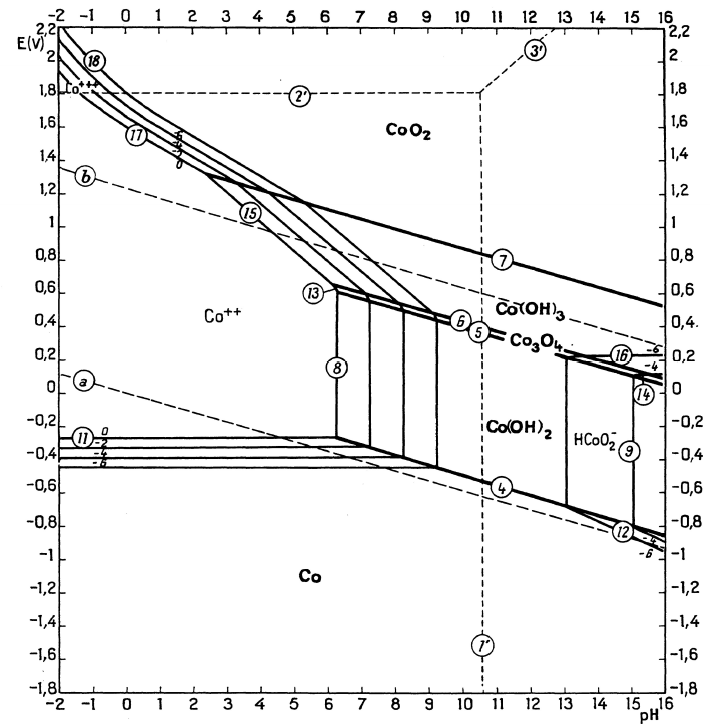


# Figure S16. Pourbaix diagram of cobalt species in an aqueous medium at 25 °C.^[8]^


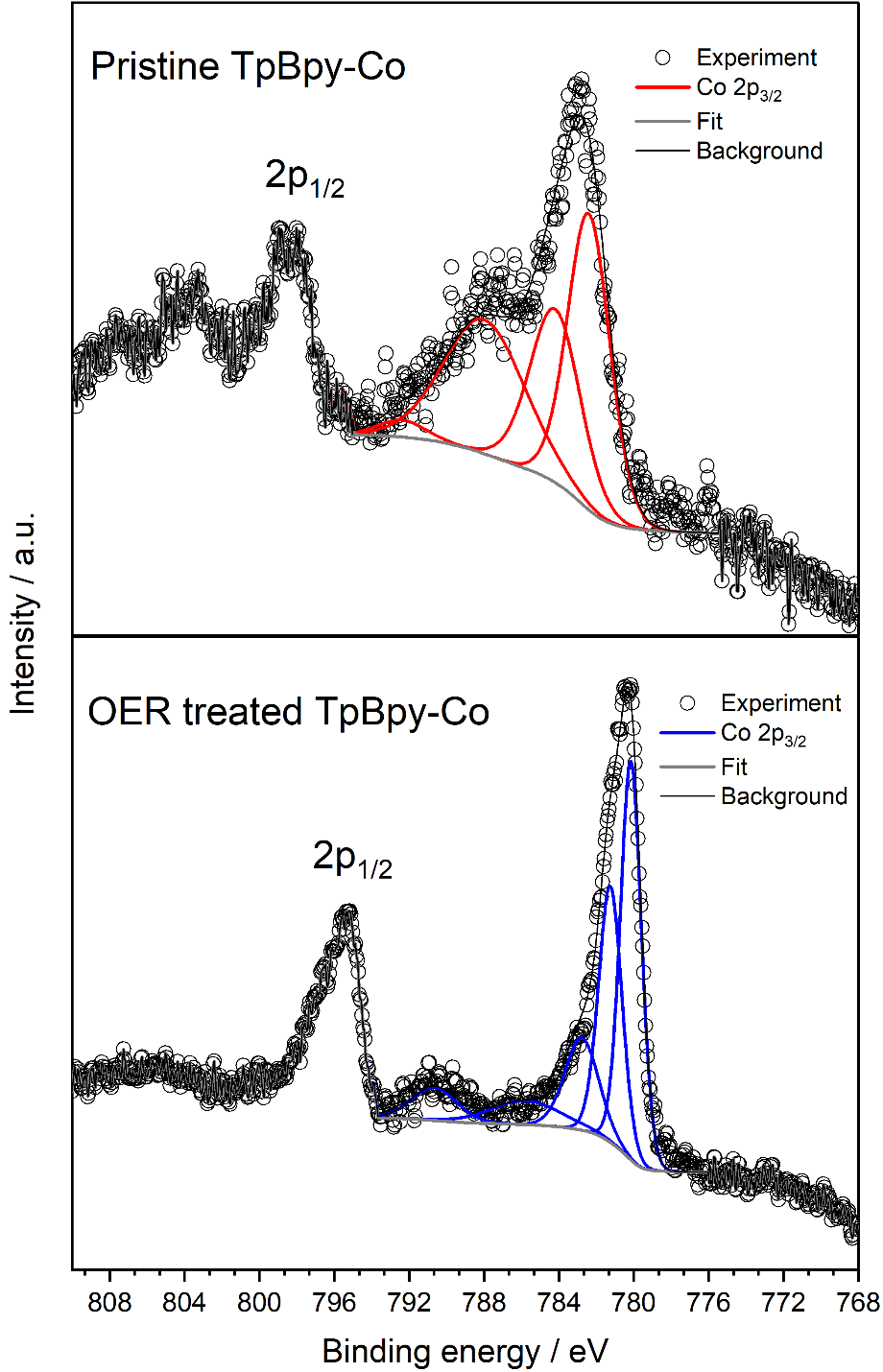


# **Figure S17.** X-ray photoelectron spectra of Co 2p of pristine TpBpy-Co and OER treated TpBpy-Co and peak fitting of Co 2p_3/2_ spectrum (open circles: experimental data, dark line: envelop and light grey line: background).

To accurately model the Co region, we followed the method outlined by Besigner et al.^[1]^ In this method, multiplet splitting was incorporated into the data fitting procedure. The peak model was constructed using reference measurements, with key fitting parameters—such as line shape, splitting values, and peak intensity ratios—constrained to the reference model, while peak positions were allowed to vary freely during the fitting process.

For the pristine sample, the optimal fit was achieved with Co²⁺ in its expected coordination environment, consistent with cobalt hydroxide. This model was characterized by four components (multiplet splitting and satellite). After the OER treatment, the best fit corresponded to Co^(II,III)^_3_O_4_, exhibiting five components (multiplet splitting and satellite), which matched the experimental data. These findings indicate a change in the chemical state of cobalt, with a partial increase in oxidation state from +2 to +3 following the OER treatment.

It is important to note that Co^3+^ cations in the initial 3d^6^ state have paired electrons, resulting in a low-spin configuration (S = 0). Therefore, no multiplet splitting is expected for Co^3+^ cations. In contrast, Co^2+^ cations in the initial 3d^7^ state possess unpaired electrons, leading to a high-spin configuration (S = 3/2). As a result, multiplet splitting is expected for Co^2+^ cations due to the coupling of the final states with the unpaired 3d electrons. This multiplet splitting causes asymmetry and broadening of the main photoemission peak.

# **Table S1.** Co 2p_3/2_ spectral fitting parameters (BE.: Binding energy, L.Sh.: line shape and FWHM: full width at half-maximum).

| Sample | BE.(eV) | FWHM | L.Sh. | %Area |
| --- | --- | --- | --- | --- |
| Pristine TpBpy-Co | 782.39 | 2.66 | GL(30) | 38.06 |
|  | 784.19 | 3.17 | GL(30) | 26.57 |
|  | 787.98 | 5.09 | GL(30) | 32.97 |
|  | 792.38 | 2.95 | GL(30) | 2.40 |
| OER treated TpBpy-Co | 780.15 | 1.31 | GL(30) | 39.00 |
|  | 781.25 | 1.47 | GL(30) | 28.03 |
|  | 782.77 | 2.13 | GL(30) | 14.64 |
|  | 785.77 | 4.39 | GL(30) | 7.80 |
|  | 790.65 | 2.82 | GL(30) | 10.53 |


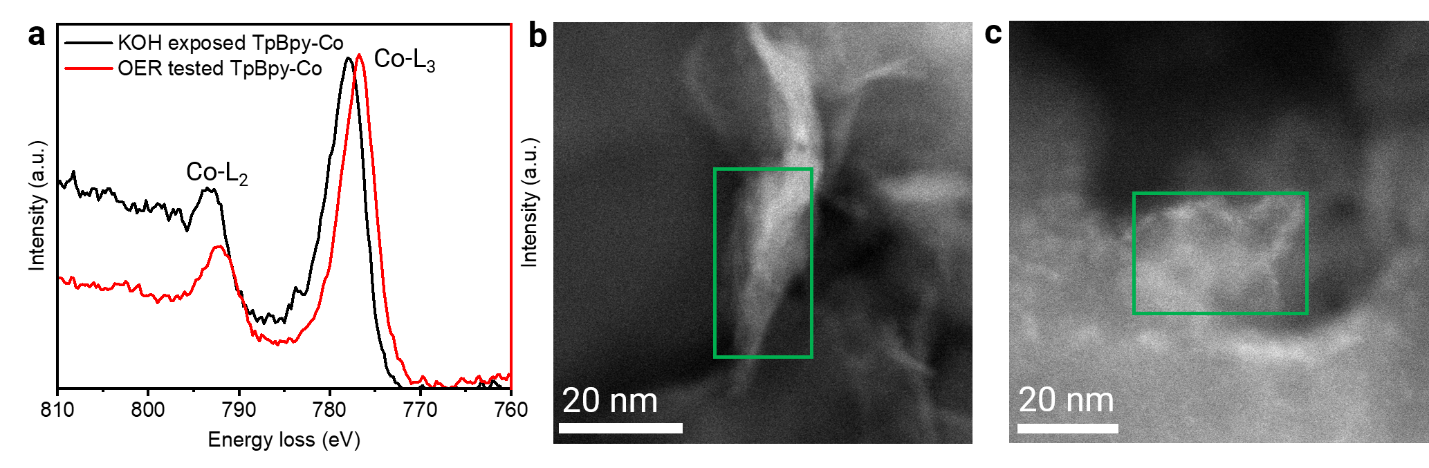


# Figure S18. EELS spectra (a) collected from the area within the green box of KOH exposed TpBpy-Co (b) and OER tested TpBpy-Co (c).

# **Figure S19.** UV-Vis absorption spectra of the EDTA/KOH solution (0.1 M Na_4_EDTA in 0.1 M KOH solution) after immersing Co(OAc)_2_, TpBpy-Co or COF-366-Co in the solution for 1 min. It can be seen that the exposure of Co(OAc)_2_ and TpBpy-Co to EDTA/KOH solution leads to the appearance of Co-EDTA complex absorption around 550 nm. In comparison, COF-366-Co does not show noticeable Co-EDTA complex absorption after immersing in EDTA/KOH solution. The results suggest that COF-366-Co coordinates the Co(II) ion more strongly.


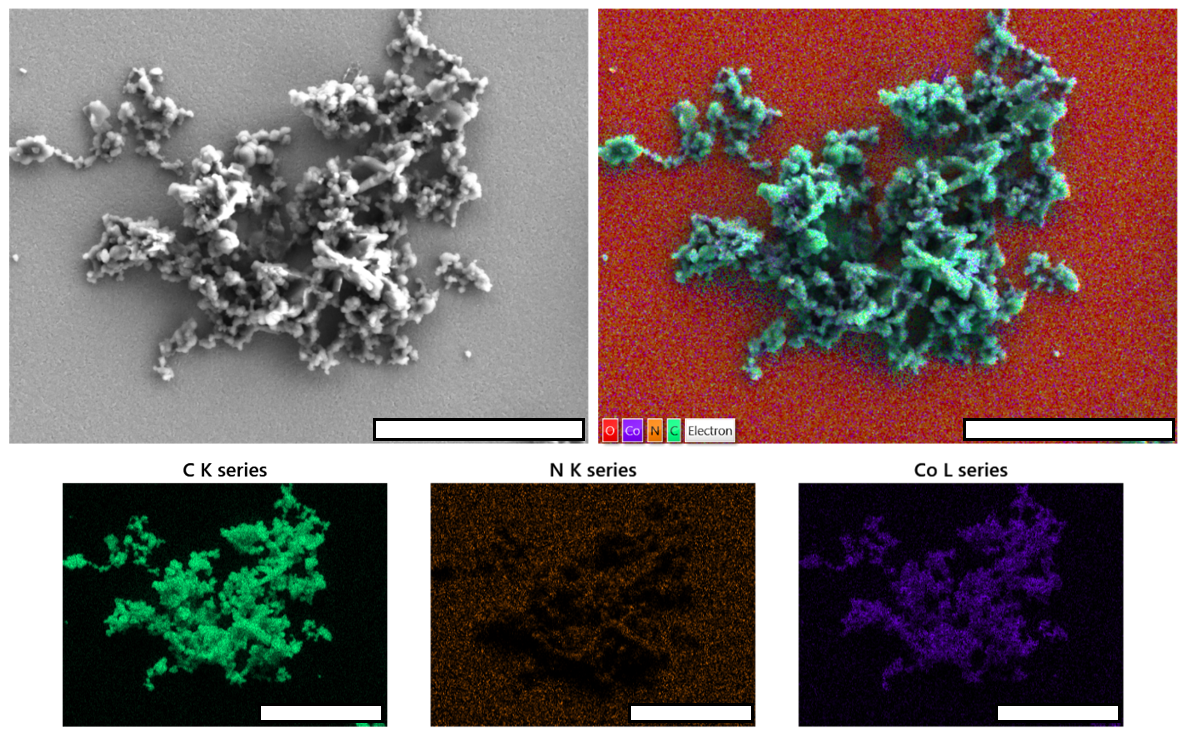


# Figure S20. SEM-EDX elemental mapping of pristine TpBz+Co(OAc)_2_ on an ITO substrate. Scale bar 10.0 μm.


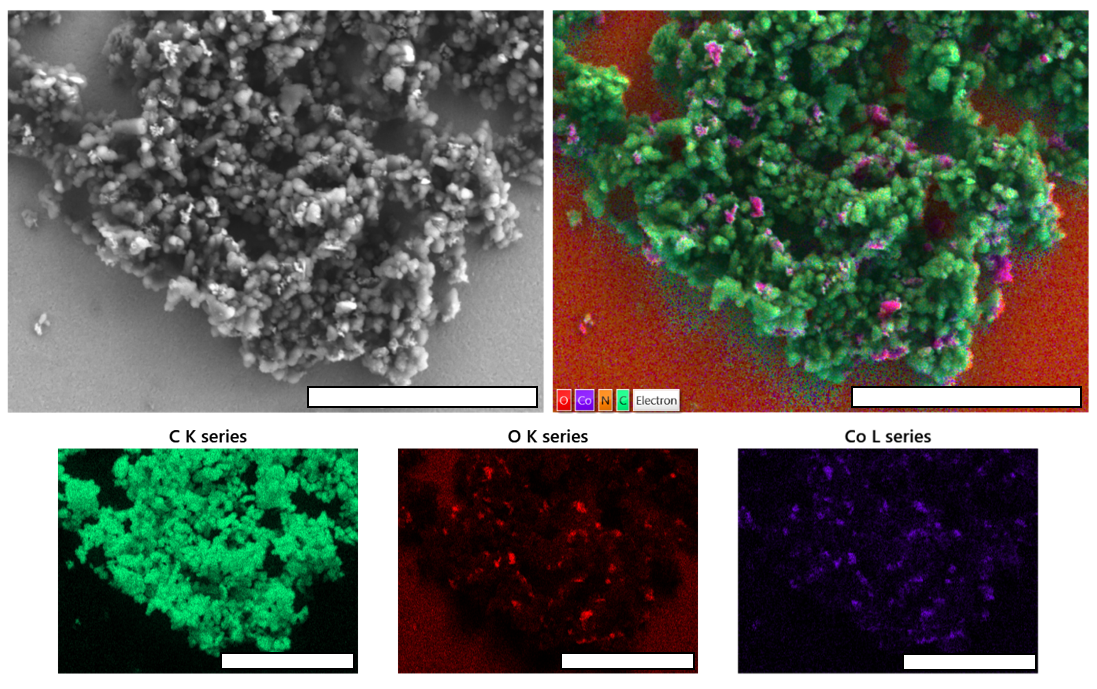


# Figure S21. SEM-EDX elemental mapping of pristine TpBz+Co(OAc)_2_ after 1-minute immersion in 0.1 M KOH pH 12.9, on an ITO substrate. Scale bar 10.0 μm.


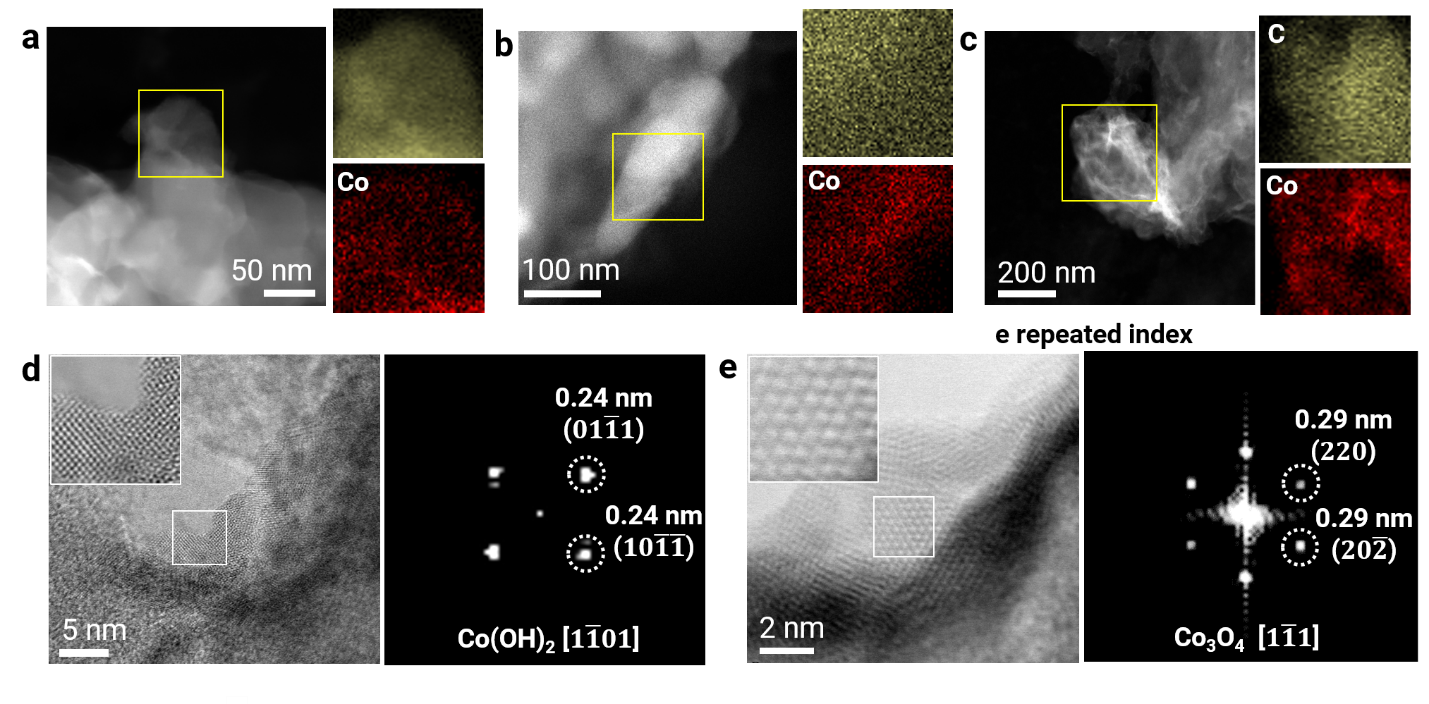


# Figure S22. STEM images of the pristine, KOH exposed, and OER tested TpBz+Co(OAc)_2_. (a-c) High angle annular dark field (HAADF)-STEM images and the corresponding EDX elemental mapping (carbon, cobalt) of pristine TpBz+Co(OAc)_2_ (a), KOH exposed TpBz+Co(OAc)_2_ (b), and OER tested TpBz+Co(OAc)_2_ (c). (d, e) High resolution bright field (BF)-STEM images with corresponding FFT patterns (labelled with crystallographic indices) from the boxed regions of KOH exposed TpBz+Co(OAc)_2_ (d) and OER tested TpBz+Co(OAc)_2_ (e).


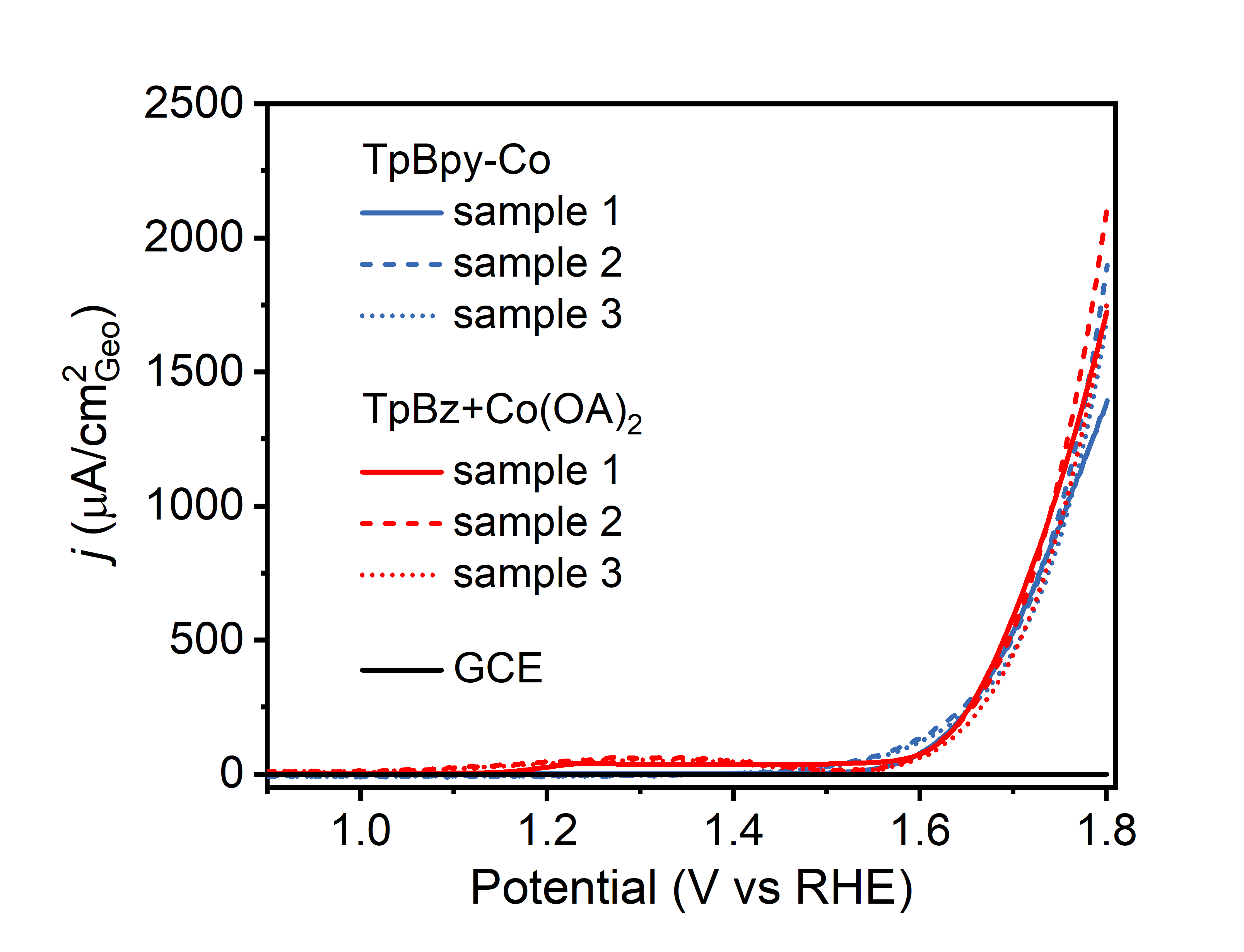


# Figure S23. Current density (geometric) – potential curves of TpBpy-Co, TpBz+Co(OAc)_2_ and GCE in 0.1 M KOH with 10 mV s^–1^ scan rate.


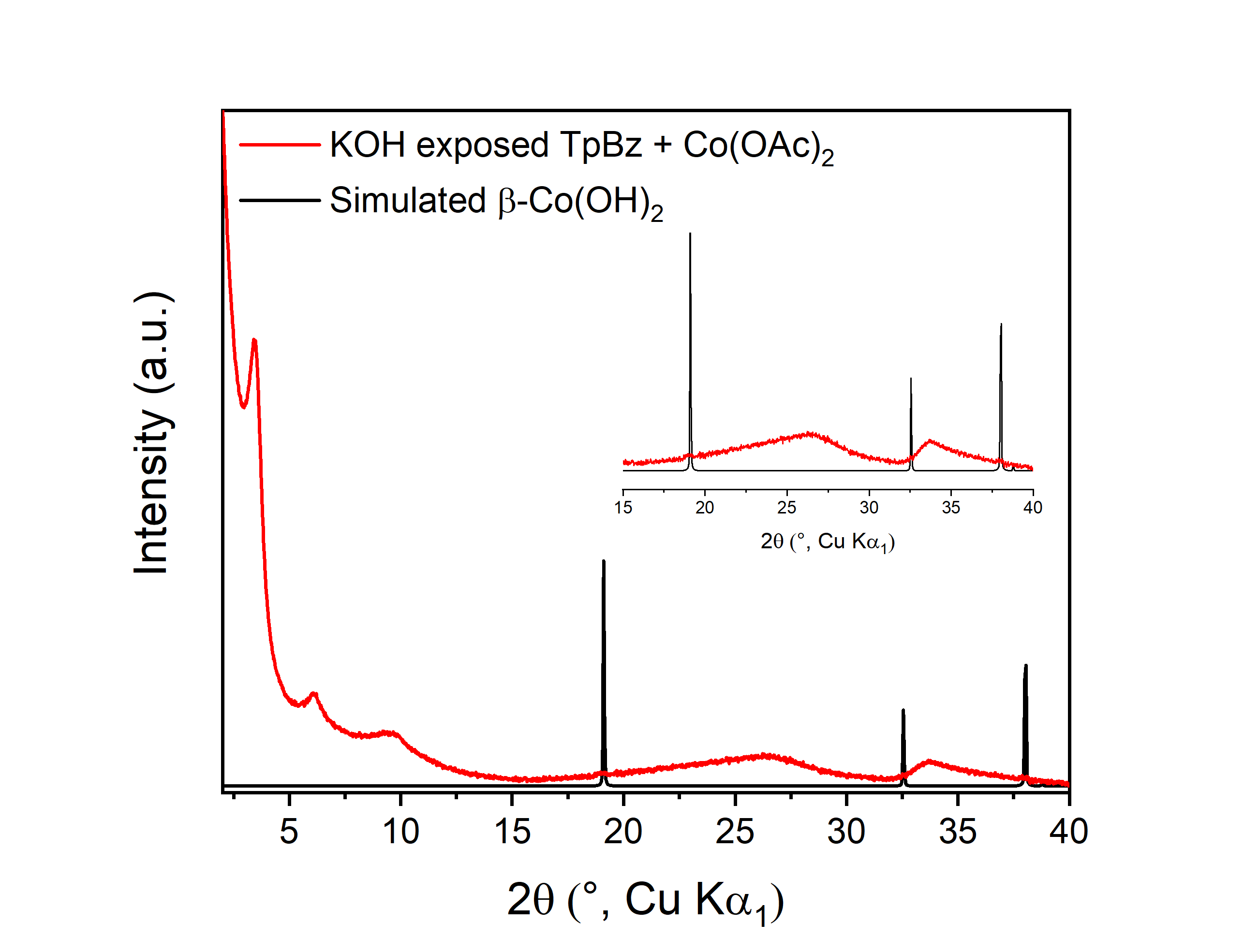


# Figure S24. Experimental PXRD pattern of KOH exposed TpBz + Co(OAc)_2_ and simulated diffraction pattern of β-Co(OH)_2_.

# References

[1] M. C. Biesinger, B. P. Payne, A. P. Grosvenor, L. W. M. Lau, A. R. Gerson, R. S. C. Smart, *Applied Surface Science* **2011**, *257*, 2717.

[2] S. Zhang, C. Scheu, *Microscopy* **2018**, *67*, i133.

[3] J. H. Chong, M. Sauer, B. O. Patrick, M. J. MacLachlan, *Organic Letters* **2003**, *5*, 3823.

[4] H. B. Aiyappa, J. Thote, D. B. Shinde, R. Banerjee, S. Kurungot, *Chemistry of Materials* **2016**, *28*, 4375.

[5] R. Wang, W. Kong, T. Zhou, C. Wang, J. Guo, *Chemical Communications* **2021**, *57*, 331.

[6] K. Endo, A. Raza, L. Yao, S. Van Gele, A. Rodríguez‐Camargo, H. A. Vignolo‐González, L. Grunenberg, B. V. Lotsch, *Adv. Mater.* **2024**, *36*, 2313197.

[7] M. Corva, N. Blanc, C. J. Bondue, K. Tschulik, *ACS Catal* **2022**, *12*, 13805.

[8] M. Pourbaix, *NACE International* **1974**, p 325.
